# Nanoscale Coatings for Ultralow Dose BMP‐2‐Driven Regeneration of Critical‐Sized Bone Defects

**DOI:** 10.1002/advs.201800361

**Published:** 2018-11-19

**Authors:** Zhe A. Cheng, Andres Alba‐Perez, Cristina Gonzalez‐Garcia, Hannah Donnelly, Virginia Llopis‐Hernandez, Vineetha Jayawarna, Peter Childs, David W. Shields, Marco Cantini, Laura Ruiz‐Cantu, Andrew Reid, James F. C. Windmill, Elena S. Addison, Sandra Corr, William G. Marshall, Matthew J. Dalby, Manuel Salmeron‐Sanchez

**Affiliations:** ^1^ Centre for the Cellular Microenvironment University of Glasgow G12 8LT Glasgow UK; ^2^ Centre for Additive Manufacturing University of Nottingham Nottingham UK; ^3^ Centre for Ultrasonic Engineering Department of Electronic and Electrical Engineering University of Strathclyde Glasgow UK; ^4^ Small Animal Hospital University of Glasgow Glasgow UK

**Keywords:** biomaterials, bone regeneration, growth factor delivery, stem cell differentiation

## Abstract

While new biomaterials for regenerative therapies are being reported in the literature, clinical translation is slow. Some existing regenerative approaches rely on high doses of growth factors, such as bone morphogenetic protein‐2 (BMP‐2) in bone regeneration, which can cause serious side effects. An ultralow‐dose growth factor technology is described yielding high bioactivity based on a simple polymer, poly(ethyl acrylate) (PEA), and mechanisms to drive stem cell differentiation and bone regeneration in a critical‐sized murine defect model with translation to a clinical veterinary setting are reported. This material‐based technology triggers spontaneous fibronectin organization and stimulates growth factor signalling, enabling synergistic integrin and BMP‐2 receptor activation in mesenchymal stem cells. To translate this technology, plasma‐polymerized PEA is used on 2D and 3D substrates to enhance cell signalling in vitro, showing the complete healing of a critical‐sized bone injury in mice in vivo. Efficacy is demonstrated in a Münsterländer dog with a nonhealing humerus fracture, establishing the clinical translation of advanced ultralow‐dose growth factor treatment.

## 1 Introduction

Active lifestyles in the young, obesity, diabetes, and osteoporosis in the elderly are driving an increase in the occurrence of traumatic injury. In 2010, >250 000 people in the USA over the age of 65 were hospitalized for hip fractures,^1^ with an increasing number of patients experiencing nonhealing (known as nonunion) fractures.^2^ Rates of nonunions from 2 to 5% have been recently suggested,^3^, ^4^ with important variations with type of fracture, age, gender, and risk factors. Nonunion fractures account for the most inpatient hospital days of all musculoskeletal injuries at a cost of $9.8 billion annually in the USA.^5^ With an ageing population, >1 million fractures are expected to occur annually by 2050.^5^ Thus, there is an urgent need to develop new bone repair therapies that are safe, cost‐effective, and efficacious.

Currently, therapeutic approaches for the treatment of nonunion fractures include growth factor (GF)‐based treatments,^6^, ^7^ stem cell therapies,^7^ and magnetic field treatments.^7^, ^8^ GFs, in particular bone morphogenetic proteins (BMPs), are commonly used in fracture treatments. However, they are not without limitations, including partial efficacy^9^; uncontrolled and nonlocalized delivery, which can produce potentially harmful, nonspecific, systemic side effects^10^, ^11^, ^12^; and high cost due to the large doses of GFs used.^9^, ^13^

BMP‐2 has been used for over a decade in bone regenerative therapies, loaded into collagen sponges at high concentrations (1.5 mg mL^−1^).^14^ Despite the US Food and Drug Administration (FDA) releasing a notification of the life‐threatening complications associated with the therapeutic use of high‐dose BMP‐2 for bone repair, including ectopic bone formation, neurological problems, and high risk of cancer, the use of GF therapies continues.^15^ Nevertheless, new advanced technologies are being developed to replace existing GF‐based treatments by exploiting the bioactive properties of materials.^16^, ^17^, ^18^, ^19^, ^20^, ^21^, ^22^ Still, the translation of materials‐based platforms from in vitro and in vivo lab testing through to clinical applications remains limited due to overengineering, use of novel chemistry unlikely to gain regulatory approval, and/or complex grafting of biologicals.

Structural scaffolds based on FDA‐approved materials can potentially be used in conjunction with bioactive polymer coatings. One such bioactive polymer is poly(ethyl acrylate) (PEA).^17^ We have previously shown that while fibronectin (FN) typically adsorbs onto polymers in globular conformation, PEA promotes the spontaneous organization of FN into physiological‐like networks. When assembled, these networks present both integrin‐binding (III_9–10_) and GF‐binding (III_12–14_) regions to cells.^17^, ^23^ Importantly, the GF‐binding region can stably present GFs, such as BMP‐2, at ultralow doses.^17^ However, the application of bioactive polymer coatings, such as PEA, to 3D scaffolds and their clinical translation have been hindered by several limitations. First, the current predominant technique for coating a surface with PEA is spin coating,^17^, ^24^ which is difficult to achieve on a 3D scaffold. Second, PEA is nonbiodegradable. One solution to this problem would be to coat a biodegradable polymeric scaffold with a layer of PEA that is thin enough (<10s of nm) for the body to metabolize after the scaffold has degraded.^25^, ^26^ Standard polymer coating techniques such as spin coating typically yield a PEA coating with a thickness of ≈1 µm on a flat surface. This is too thick for clinical purposes, even if spin coating was to be successfully implemented on a 3D scaffold. Spin coating also requires PEA to be dissolved in an organic solvent and thus traces of harmful solvents can remain. We therefore sought to develop a solvent‐free coating method^24^ with nanoscale coating depth that could be applied to a variety of 3D applications, such as scaffolds and grafts.

To achieve this, we present a simple, robust, and translational approach to promoting bone regeneration in nonunion bone defects. We report the development of a plasma–polymerization strategy for coating 3D scaffolds with thin layers of PEA. We show that this highly facile method can be used to coat 2D surfaces and complex 3D scaffolds, including 3D‐printed scaffolds and microparticles, and that this coating can assemble FN in the form of biomimetic networks. We investigate whether these FN networks support synergistic interactions between integrins and BMP‐2 receptors that promote osteogenesis in vitro. Importantly, this nanoscale coating can be applied to 3D biodegradable materials and we investigate their use as an implant to advance bone healing in a mouse model of a critical‐sized, nonhealing bone defect. We also report the first veterinary application of this technology to treat a 2‐year‐old Münsterländer dog with a nonhealing and infected fracture of the humerus. We treat this nonunion using PEA‐coated decellularized bone chips seeking to promote rapid and robust healing of this bone defect and prevent limb amputation. Together, we introduce a next‐generation GF‐based technology for effective bone repair in vivo that is likely safer in a clinical setting given the ultralow dose of BMP‐2 used.

## 2 Results

### 2.1 Plasma‐Polymerized PEA Coatings Promote FN Assembly and Effective BMP‐2 Presentation

**Figure 1:**
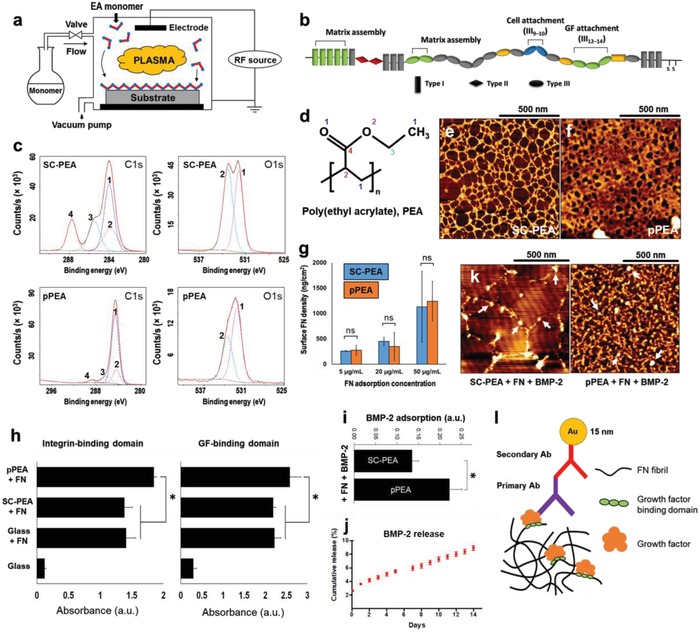
Physicochemical characterisation of plasma PEA coatings, FN, and BMP‐2 adsorption on pPEA. a) Schematic representation of a custom‐made plasma polymerization chamber. b) FN structure, showing its three domain types (I, II, III) and functions. Domain III region (III_9–10_) contains the RGD (Arg‐Gly‐Asp) sequence that facilitates cell adhesion via integrin binding, and region III_12–14_ binds various GFs, including BMP‐2. c) XPS characterization of SC‐PEA and pPEA. High‐resolution C1s and O1s spectra are shown with fitted components in colored dotted lines. d) Chemical structure of PEA, with labelled carbon and oxygen atoms corresponding to components in panel (c). AFM phase images of FN adsorbed for 10 min on e) SC‐PEA and f) pPEA. Thin fibrillar networks were observed on SC‐PEA, whereas thick, dense networks were observed on pPEA. g) Surface density of FN adsorbed at different concentrations onto SC‐PEA and pPEA for 1 h. h) Relative exposure of integrin‐binding and GF‐binding domains on FN adsorbed on different surfaces, measured using ELISA. i) Relative adsorption of BMP‐2 on FN‐coated surfaces, measured using ELISA. j) Cumulative BMP‐2 release from surfaces coated with pPEA, FN, and BMP‐2 during 2 weeks. k) AFM height images of BMP‐2 labelled with gold nanoparticles on SC‐PEA and pPEA coated with FN. White arrows indicate gold nanoparticles showing BMP‐2 distribution. l) Schematic representation of immunogold assay for GF detection. All data are presented as mean ± SD, *n* = 3, one‐way ANOVA with Tukey's test for multiple comparisons. *p < 0.05, ns = not statistically significant.

The plasma‐based polymerization of ethyl acrylate into plasma‐polymerized PEA (pPEA) coatings is illustrated in **Figure** **1**a. We used spin‐coated PEA (a nontranslatable approach, as explained above), denoted as SC‐PEA, as a positive control. When FN is coated on SC‐PEA (open FN chain, illustrated in Figure 1b), BMP‐2 binding, crosstalk with integrins, and osteogenesis are promoted, as previously demonstrated.^17^ The chemistry of pPEA was confirmed using X‐ray photoelectron spectroscopy (XPS). The spectrum of pPEA shows a prominent disappearance of the C—O and C—O—C moieties at 286.6 and 533.5 eV, respectively, which differs from that of SC‐PEA (Figure 1c,d).^27^ It is known that plasma polymerization leads to partial loss of functional groups and crosslinking.^24^ By fine‐tuning the parameters of the plasma system, such as chamber pressure, power, and polymerization time, we were able to coat pPEA onto glass at a rate of as low as 10 nm min^−1^ (Figure S1a, Supporting Information). The thickness of the deposited pPEA coatings (≈300 nm) guarantees that the XPS spectra are due to pPEA and not to the underlying substrate, as the sensitivity of XPS is ≈10 nm.^24^ The stability of the films was confirmed by monitoring the water contact angle (WCA) after deposition and up to 14 d. Figure S1b in the Supporting Information shows that WCAs remain unchanged at different time points after deposition (Figure S1b, Supporting Information). We measured the strength of interaction between pPEA coatings and the underlying substrates using a normalized pull‐off test that resulted in an adhesion strength of 7.14 ± 0.35 MPa (Figure S1c, Supporting Information). Changes in plasma settings resulted in coatings of different thicknesses but with similar XPS spectra, indicating that the properties of the pPEA deposited at different conditions were comparable (Figure S2, Supporting Information).

Atomic force microscopy (AFM) was performed to visualize the organization of FN on both SC‐PEA and pPEA (Figure 1e,f). Poly(methyl acrylate) (PMA), which has one fewer carbon in its side chain, is a good control for PEA as it results in FN adsorption in globular conformation.^28^, ^29^, ^30^ (Data on interactions with PMA are presented in Figure S3a,b in the Supporting Information.) FN assembly into nanonetworks on SC‐PEA (Figure 1e) has been well characterized previously.^28^, ^29^ It occurs when FN is adsorbed onto SC‐PEA at a sufficiently high concentration (>10 µg mL^−1^) so that when FN unfolds, it can contact neighbouring FN molecules. This allows nanonetworks to assemble via interactions among FN matrix‐binding regions. AFM showed that pPEA coatings do indeed induce this FN assembly but with denser, thicker morphology compared with those formed on SC‐PEA (Figure 1f and Figure S3c, Supporting Information), although the measured surface densities of FN adsorbed on pPEA and SC‐PEA were similar (Figure 1g and Figure S3d, Supporting Information). Note that spin‐coated PMA (Figure S3e, Supporting Information) did not induce fibrillogenesis (Figure S3a,b, Supporting Information).

To assess the activity of FN on pPEA, we quantified the availability of the integrin‐binding and GF‐binding regions using enzyme‐linked immunosorbent assay (ELISA) with monoclonal antibodies against the respective regions (Figure 1h). We looked at the FN(III_9–10_) domain, which includes the RGD (Arg‐Gly‐Asp) sequence for integrin binding,^30^, ^31^, ^32^ and the FN(III_12–14_) domain,^17^ which binds a variety of GF families including BMP‐2.^16^, ^23^ The availability of both domains was significantly higher on pPEA coatings than on SC‐PEA surfaces (*p* < 0.05). Given that we normalized the graphs to total surface FN (Figure 1g and Figure S3f, Supporting Information), we propose that the reported differences are not due to the total amount of FN adsorbed on the surface (as the densities are similar between SC‐PEA and pPEA), but instead to the enhanced availability of specific binding sites on FN with pPEA.

Next, we used ELISA to quantify the amount of BMP‐2 adsorbed on FN‐coated surfaces (on SC‐PEA and pPEA coatings) from a solution at a concentration of 50 ng mL^−1^ BMP‐2 (Figure 1i); this is the concentration used in subsequent biological experiments. We also assessed the stability of the adsorbed BMP‐2 on FN‐coated pPEA and quantify the release from the surface as a function of time. More than 90% of the adsorbed BMP‐2 remains in the surface after 14 d. (Figure 1j). In line with the above data on GF binding‐site availability, more BMP‐2 was adsorbed on FN coated on pPEA than on SC‐PEA (Figure 1i and Figure S4, Supporting Information). To assess the spatial distribution of BMP‐2 molecules on the FN nanonetworks, immunogold staining was performed on SC‐PEA‐ and pPEA‐treated surfaces coated with different concentrations of FN and exposed to 25 ng mL^−1^ BMP‐2 (Figure 1k). The localization of BMP‐2 is indicated by bright dots on the AFM images, corresponding to gold nanoparticles with a diameter of ≈15 nm conjugated to primary antibodies specific for BMP‐2 (Figure 1l). Gold nanoparticles indicative of BMP‐2 (Figure 1k) interact specifically with individual nanofibers of FN on both SC‐PEA and pPEA and confirm the specific localization of BMP‐2 on FN. (Note that lower concentrations of FN and BMP‐2 than those used in cell experiments were used here to clearly depict this localization.)

### 2.2 Plasma‐Polymerized PEA Coatings Drive Synergistic Signaling and hMSC Osteogenesis

To investigate whether pPEA‐induced BMP‐2 presentation drives cell adhesion, enhances synergistic integrin/GF signalling, and is osteoinductive at low GF concentration, we cultured human mesenchymal stem cells (hMSCs) on pPEA surfaces first coated with FN and then 50 ng mL^−1^ BMP‐2.

First, cell attachment and spreading after 24 h were significantly enhanced on pPEA‐coated surfaces with FN compared to cells on SC‐PEA‐coated surfaces with FN (Figure S5, Supporting Information), as evidenced by the higher number of attached cells, greater degree of spreading, and enhanced focal adhesion (FA) formation. This correlated well with the better availability of FN(III_9–10_) domains on pPEA (Figure 1h).

**Figure 2:**
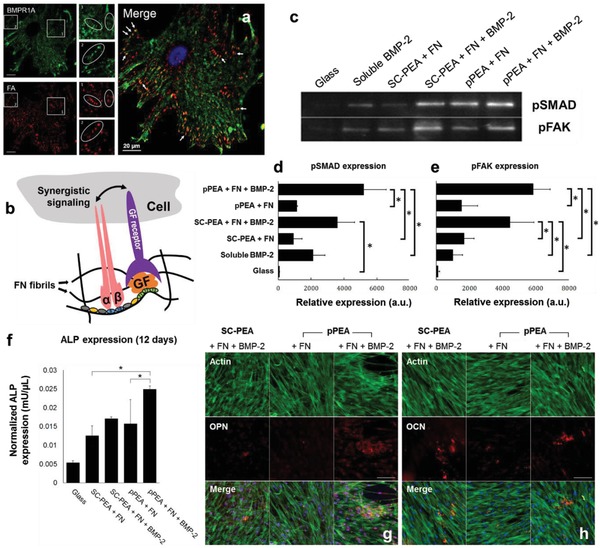
hMSC signalling and differentiation. a) Colocalization assay of BMP receptor 1A (BMPR1A, green) and FAs (red). White arrows on the merged image show areas of colocalization in yellow. Scale bar = 20 µm. b) A schematic representation showing that synergistic signalling between integrin and GF receptors can occur when the integrin‐binding (III_9–10_) and GF‐binding (III_12–14_) domains of FN are in close proximity. c) Western blotting of pSMAD 1/5/9 and pFAK, expressed by hMSCs after 1 h in culture on SC‐PEA and pPEA, with and without FN and BMP‐2. Quantified blots to show relative expressions of d) pSMAD and e) pFAK, both normalized using total protein amount, from hMSCs after 1 h in culture on SC‐PEA and pPEA, with and without FN and BMP‐2. hMSCs cultured with soluble BMP‐2 and on glass alone were used as controls. Data are presented as mean ± SD, *n* = 3, one‐way ANOVA with Tukey's test for multiple comparisons. *p < 0.05. f) Normalized ALP expression in hMSCs after 12 d in culture on SC‐PEA and pPEA surfaces, with and without FN and BMP‐2, from a fluorescent ALP assay. Data are presented as mean ± SD, *n* = 3, one‐way ANOVA with Tukey's test for multiple comparisons. *p < 0.05. Immunofluorescent labelling of g) OPN and h) OCN in hMSCs cultured on SC‐PEA and pPEA, with and without FN and BMP‐2, for 21 d. Phalloidin stains actin cytoskeleton in green and DAPI stains nuclei in blue. Scale bar = 50 µm.

Next, synergistic integrin and GF signalling were evaluated, initially by assessing whether BMP receptor 1A (BMPR1A) colocalizes with focal adhesions. Vinculin (which stains focal adhesions) immunofluorescence colocalized with BMPR1A immunofluorescence in single cells (**Figure** **2**a), indicating that BMPR1A and integrins are in close proximity, which would enable crosstalk to occur between the adhesion and GF pathways (Figure 2b). (The staining of specific integrins is shown in Figure S6 in the Supporting Information.) Next, the phosphorylation of small mothers against decapentaplegics (SMAD) and focal adhesion kinase (FAK) was examined in order to investigate BMP‐2‐related signalling and focal adhesion‐related signalling pathways, respectively.^33^, ^34^, ^35^ The expression of phosphorylated SMADs (pSMAD) and phosphorylated FAK (pFAK) in hMSCs after 1 h of culture on the test versus control surfaces is shown in Figure 2c.

SMADs 1, 5, and 9 can be phosphorylated by BMPR1A, leading to their nuclear translocation and the activation of RUNX2 (runt‐related transcription factor 2, the osteogenic master transcription factor).^33^, ^36^ Quantification of pSMAD expression by western blotting, normalized by total protein amount (Figure 2d and Figure S7, Supporting Information), showed a significant upregulation of pSMAD on pPEA‐coated surfaces with FN and BMP‐2 (pPEA + FN + BMP‐2) compared with pPEA without BMP‐2 (pPEA + FN); a similar trend was observed on SC‐coated surfaces (Figure 2d). In both cases, the presentation of BMP‐2 on PEA resulted in enhanced SMAD signalling compared to the soluble administration of the GF alone (soluble BMP‐2, Figure 2d).

We also examined the expression of pFAK in hMSCs, which was normalized using total protein amount (Figure 2e and Figure S7, Supporting Information). pFAK is involved in integrin‐related signalling^35^, ^37^ and was significantly upregulated (*p* < 0.05) in hMSCs cultured on surfaces coated with pPEA + FN + BMP‐2 relative to those cultured without BMP‐2 (pPEA + FN) or with soluble BMP‐2 alone (again, a similar trend was observed for the control material, SC‐PEA). We note that in hMSCs cultured with soluble BMP‐2, pFAK is induced to the same level as it is in cells cultured on pPEA without BMP‐2, which suggests that increased pFAK expression is focal adhesion related and occurs independently of BMP‐2. From these findings, we propose that enhanced synergistic adhesion and BMP‐2 signalling on pPEA + FN + BMP‐2 occurs as a consequence of the simultaneous occupancy of integrins and BMP‐2 receptors.^35^, ^37^

We next investigated the potential of this synergistic signalling cascade, driven by PEA‐induced assembly of FN, for osteogenesis. Alkaline phosphatase (ALP) expression was measured after 12 d of hMSC culture (Figure 2f). ALP, which plays a role in bone mineralisation and is an early marker of osteogenic differentiation,^38^ was expressed by hMSCs cultured on pPEA + FN + BMP‐2 at a significantly higher level than it was by cells cultured on pPEA + FN without BMP‐2 coating. Long‐term culture was also performed for 21 d and two osteogenesis‐related proteins, osteopontin (OPN) and osteocalcin (OCN), were visualized by immunofluorescence (Figure 2g,h and Figure S8, Supporting Information). Both OPN and OCN (shown in red) were more highly expressed in hMSCs cultured on pPEA + FN + BMP‐2 than they were by cells cultured on pPEA‐FN surfaces without BMP‐2. Moreover, comparing SC‐PEA and pPEA, it is clear that when both FN and BMP‐2 were present, OPN was expressed more distinctly on the pPEA‐coated surfaces. Von Kossa staining further revealed the presence of mineralized deposits on the surfaces coated with BMP‐2 (Figure S9, Supporting Information).

As proof of concept that pPEA can be employed as a coating for 3D biomaterials, further in vitro analysis was performed on 3D polycaprolactone (PCL) scaffolds (cylinders of 5 mm diameter, 800 µm high). The scaffolds were fabricated by 3D printing (to generate filaments of 200 µm in diameter, with 500 µm separation, layered at 90° to the last layer, see Figure S10a in the Supporting Information). Once fabricated, the scaffolds were coated with pPEA for 30 min before adsorbing FN to generate a fibrillar network (inset, Figure S10a, Supporting Information). FN adsorption was confirmed by ELISA (Figure S10b, Supporting Information). The expression of BMPR‐2, RUNX2, and osterix (OSX) was then quantified by quantitative polymerase chain reaction (qPCR) (Table 1, Supporting Information). qPCR results showed that by day 7 in culture, OSX expression (but not that of BMPR‐2 and RUNX2 at this time point) was significantly increased in hMSCs cultured on PCL + pPEA + FN + BMP‐2, compared to cells cultured on PCL + pPEA or on PCL + pPEA + FN (Figure S10c, Supporting Information). It is likely that BMPR‐2 and RUNX2 upregulation had occurred at an earlier time point (previous reports have shown that BMPR‐2 levels are highest at day 3, and RUNX2 levels highest at day 5 of culture, while OSX expression occurs later at days 7–11^39^). With respect to RUNX2, a significant increase in its phosphorylation was seen in PCL + pPEA + FN + BMP‐2 as early as day 5 in culture, relative to other controls (PCL, PLC + pPEA, PCL + pPEA + FN) (Figure S10d, Supporting Information). After longer‐term culture (21 d), mature bone nodules could be observed by Alizarin red staining on the scaffolds coated with PCL + pPEA + FN + BMP‐2 (Figure S10e, Supporting Information).

### 2.3 Plasma‐Polymerized PEA Coatings Drive Regeneration in a Murine Nonhealing Radial Bone Defect Model

To investigate the translational potential of the osteogenic pPEA coatings, we used an adult mouse model of a critical‐sized, nonhealing radial bone defect.^17^ This bone repair model has significant advantages: i) the 2.5 mm defect does not spontaneously heal, providing a rigorous critical‐sized model, ii) it allows for simple in vivo imaging approaches (e.g., microcomputed tomography, µCT), and iii) the ulna provides sufficient stabilization of the defect and no fixation plates/hardware are required. This simplifies the surgical procedure and reduces the risk of infection, a major advantage over the rat calvaria and segmental femur defect models.^21^

Polyimide tube implants of 4 mm were used in this model as they form a biocompatible but nonbioactive scaffold that fits well over the defect (Figure S11, Supporting Information). These were coated with degradable PCL by solvent casting, creating a layer of an FDA‐approved biomaterial within the tube. This was followed by polymerization of a very thin layer of pPEA (estimated at <100 nm) to cover the underlying PCL polymer. Then, FN or FN + BMP‐2 was adsorbed on the cylindrical polymer surface. BMP‐2 was used at low concentration, i.e., ≈15 ng of BMP‐2 on the wall of the coated tubes (surface density of 100 ± 8 ng cm^−2^, as measured using ELISA).^17^ This BMP‐2 concentration is at least 100% lower than that used in advanced materials systems previously tested in murine models that are based on integrin‐specific polyethylene glycol hydrogels loaded with BMP‐2.^21^ Note that even if humans and rodents do not metabolize biologics at the same rate, the amount of BMP‐2 used was ≈300 fold lower than that of the clinical gold standard.^14^, ^17^ Implant tubes without BMP‐2 coated only with the PCL layer or with PCL + pPEA were used as negative controls. FN and BMP‐2 adsorbed on solvent‐casted PEA were used as positive controls based on previous data.^17^

**Figure 3:**
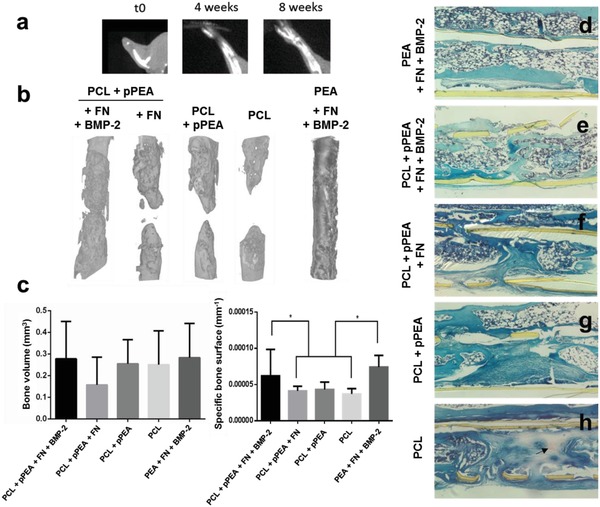
Bone regeneration in a murine model of a critical‐sized radial bone defect with low doses of BMP‐2. a) X‐ray images at 0, 4, and 8 weeks after surgery. b) 3D reconstructions from the µCT images showing the radius in the area of the defect, 8 weeks after introduction of the PCL‐pPEA implant (with or without FN and BMP‐2). c) Quantification of the volume and specific surface of new bone. Data are presented as mean ± SD, minimum *n* = 3. Two‐tailed t‐test was used to analyze data. *p < 0.1. d–h) Hematoxylin‐Safranin O‐fast green staining of histological sections in the area of the defect. The tissue is organized in structures resembling bone marrow (rounded white structures in panels (d) and (e)) versus fibroblast‐like morphology (extended and aligned) in the center of the defect in panels (f)–(h). Arrow points to red staining that indicates cartilage formation in panel (h).

Bone formation was evaluated by X‐ray (**Figure** **3**a) and by 3D µCT reconstructions, which displayed the total length of the radius scanned (Figure 3b). The 3D µCT images showed increased bone growth along the defect in the presence of BMP‐2, to the extent where the bone gap is fully bridged (Figure 3b). These results show that higher levels of bone regeneration occurred in the presence of BMP‐2, even when it was only applied to the walls of the implant tube, as performed here. Most importantly, similar levels of bone regeneration are seen on samples coated with solvent‐casted PEA (positive control) and on the test material pPEA, which indicates enhanced in vivo effects of pPEA, even with the use of low doses of BMP‐2 (Figure 3b). Further, limb function was restored in all treated mice. The quantification of bone formation from µCT images shows enhanced bone surface density in the presence of low‐dose BMP‐2 (both on PEA and pPEA) compared to conditions where no BMP‐2 was present in the implant (Figure 3c), albeit the same bone volume was found in all other conditions tested (Figure 3c). The discrepancy between bone volume and bone surface density between groups is a reflection of how bone is organized within the defect, allowing or not full bridging. A way to measure the architecture of new bone is by analyzing the 3D information provided by µCT and quantify the total surface of bone formed. This is particularly relevant for areas of the implant functionalized with BMP2, as a new layer of bone grows in contact with them. These results suggest the different organizations of bone, leading to the full bridging of defects in the pPEA + FN + BMP‐2 condition (Figure 3b).

The histological sections in the area of the defect support the findings obtained from the µCT images. Implant tubes (solvent‐casted PEA and pPEA) coated with FN and BMP‐2 induced higher levels of new bone formation, relative to controls (Figure 3d,e). Both proximal and distal bone extended into the implant, and structures that resemble the bone marrow were observed along the entire defect (Figure 3d,e). However, a larger gap between proximal and distal bone was observed in the absence of BMP‐2 (Figure 3f,g), with less bone healing observed for PCL‐only control implants (Figure 3h). Moreover, fibroblast‐like structures were observed filling the gap of the defect in PCL‐only control implants, with cartilage tissue present (red staining in Figure 3h), indicating the differentiation of cells toward the chondrogenic lineage.

Taken together, the in vitro and in vivo data indicate that the synergistic integrin/BMP‐2 receptor osteoinductive response is generated by pPEA‐coated scaffolds. From these findings, we propose this polymerization approach as a potential method for coating material implants in 2D and 3D scaffolds.

### 2.4 Veterinary Case Study: pPEA Supports Healing of a Nonunion Humeral Fracture in a Dog

**Figure 4:**
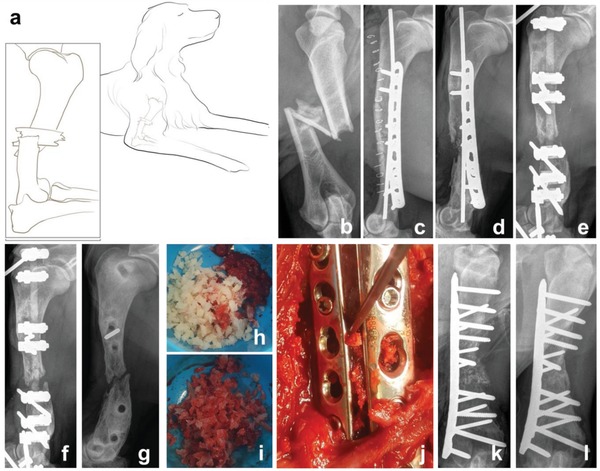
Humeral fracture healing in a dog treated with bone chips coated with pPEA, FN, and BMP‐2. a) A schematic representation of a 2‐year‐old female Münsterländer dog, showing a comminuted fracture of the diaphysis of her right humerus, sustained when she was hit by a car. b) Radiograph of the comminuted fracture of the diaphysis of the right humerus. c) Radiograph showing the surgical stabilisation of the fracture using standard open reduction and internal fixation technique. d) Five months after c), osteolysis at the fracture site was evident, consistent with osteomyelitis and delayed union. e) Restabilization of the fracture using an external skeletal fixator. f) Six weeks after (e), there was no evidence of fracture healing. g) Radiograph showing fracture nonunion, 8 months after the injury. h) Prior to surgery, decellularized bone chips were coated with pPEA to form pPEA‐chips, which were subsequently coated with FN and BMP‐2. i) Bone marrow was harvested from the humeral head on the left side and mixed with 5 cc of coated pPEA‐chips. j) Bone plates and screws were used to stabilize the fracture, and the combined graft materials were placed within the fracture gap. k) Postoperative radiograph shows the fracture gap filled with graft. l) Evidence of fracture union 7 weeks after surgery performed in panels (g) to (j).

To demonstrate the translatable potential of the pPEA system, we performed a first trial of this approach in a veterinary case study. A 2‐year‐old female Münsterländer dog (**Figure** **4**a and Figure S12 and Video S1, Supporting Information) was presented to the Small Animal Hospital, University of Glasgow, in July 2016 for the management of a comminuted fracture of the diaphysis of the right humerus, sustained when she was hit by a car (Figure 4b). The fracture was stabilized surgically using a standard open reduction and internal fixation technique (Figure 4c). The dog made a slow early recovery, retaining a significant degree of lameness and developed a discharging sinus on the medial aspect of the distal humerus around 2 months postoperatively, indicative of an infection at the fracture site. This was confirmed when *Staphylococcus spp*. was cultured from this sinus. The infection was treated with antibiotics (potentiated amoxicillin, 500 mg by mouth three times daily) for 6 weeks based on the sensitivity profile. Despite this, the dog remained persistently lame. Five months after surgery, there was no convincing evidence of fracture healing. Radiographically, areas of osteolysis and implant loosening indicated the presence of osteomyelitis and delayed union (Figure 4d).

We removed the implants on the basis that a bacterial biofilm was likely covering them and perpetuating the infection. The fracture was restabilized with an external skeletal fixator (Figure 4e) and antibiotic treatment continued (potentiated amoxicillin, 500 mg by mouth twice daily) for a further 2 weeks, as *Staphylococcus spp*. cultured directly from the metal implants showed the same sensitivity profile as previously shown.

Radiographs taken 6 weeks after revision surgery showed no evidence of fracture healing and significant osteolysis (Figure 4f). We were concerned that this fracture was developing an atrophic nonunion, and radiographs taken 10 weeks postrevision (8 months following the original injury) were consistent with this (Figure 4g). Because of the poor prognosis, we considered limb amputation but instead elected to revise the fracture fixation once more using allograft microparticles coated with pPEA on which FN and BMP‐2 were adsorbed at 50 µg mL^−1^. This concentration is 30‐fold lower than that used in clinical standards (BMP‐2 loaded in a collagen sponge at a concentration of 1.5 mg mL^−1^).^14^ We note that while this is a higher concentration than that used in our in vivo mouse bone defect model, it is still only 10% of the dose (0.5 mg mL^−1^) typically used in complex fractures in dogs in veterinary applications.^40^

Decellularized bone chips (2–4 mm) were coated with pPEA using plasma polymerization to form pPEA‐chips and subsequently coated with FN and BMP‐2 (Figure 4h). A standard medial approach was made to the humerus. Approximately 1 cm of the nonhealing ends of the fracture was excised, along with all soft issue within the fracture gap. Bone marrow was harvested from the humeral head on the left side and mixed with 5 cc of pPEA‐chips (Figure 4i). The fracture site was thoroughly debrided and then stabilized using two bone plates and screws and the combined graft materials were placed within the fracture gap (an ≈2 cm defect) (Figure 4j); soft tissues were closed as per routine protocol. Postoperative radiographs showed the fracture gap filled with graft and the appropriate placement of the implant (Figure 4k). The dog made a good recovery from the procedure.

There was a moderate degree of postoperative swelling of the affected limb, which resolved within a week, but no other adverse effects were observed. The tissue removed from the fracture site was submitted for bacterial culture, which showed the persistence of a *Staphylococcus* infection. Antibiotics were prescribed (cephalexin 300 mg by mouth twice daily) and continued for 4 months postoperatively. The dog's use of the affected limb steadily improved, and radiographs taken 7 weeks after surgery were consistent with fracture union (Figure 4l). By 6 months postoperatively, the dog had resumed normal exercise, and there was no clinical evidence of recurring infection.

## 3 Discussion

Notwithstanding significant off‐target effects, BMPs have been used in patients for the management of nonunion fractures where traditional approaches have failed.^14^ Here, we present a simple and facile technology that allows materials with complex geometries to be coated with ultralow and thus safer levels of BMP‐2 for use in bone regeneration‐promoting therapies to treat critical‐sized, nonunion bone defects. Plasma polymerization presents a solvent‐free coating method that can be used for a variety of 3D applications, such as scaffolds and grafts.

This new approach produces thin PEA coatings (layers of 30–200 nm are possible) which are strongly attached to the underlying substrate (≈7.10 MPa – Figure S1, Supporting Information) that can assemble FN in the form of biomimetic nanonetworks (Figure 1f), rather than as a globular structure. This unfolding of the FN molecule via nanonetwork formation exposes sites responsible for FN function, such as integrin‐binding (III_9–10_) and GF‐binding (III_12–14_) regions.^17^, ^23^, ^30^, ^31^

In in vitro assays, synergistic signalling was observed in hMSCs cultured on surfaces coated with pPEA + FN + BMP‐2, together with the upregulation of pSMADs (Figure 2c,d), indicating the onset of canonical BMP‐2 signalling mediated by BMP receptors, as shown previously.^17^ Increased pFAK with BMP‐2 stimulation from the surface but not in soluble form indicates integrin ligation.^35^ Previous research has shown that intact integrin function is crucial for BMP‐2 activity and that inhibition of FAK activation blocks SMAD signalling activation by BMP‐2.^37^ The upregulation of pFAK on BMP‐2‐coated surfaces is shown in Figure 2c,e, signifying that the combined effects of pSMAD and pFAK signalling could be a consequence of the potency of BMP‐2 in enhancing downstream osteogenic activities. Previous evidence has demonstrated that crosstalk occurs between GF receptors and integrins, which plays a crucial role in regulating mechanotransduction in the extracellular matrix.^17^, ^37^, ^41^, ^42^, ^43^ The colocalization of BMP‐2 receptors with various types of integrins has been demonstrated previously,^17^, ^37^, ^39^ and this colocalization is confirmed in our study (Figure 2a).

We show that pPEA + FN + BMP‐2 promotes bone regeneration in a critical‐sized defect in the mouse radius. The effect is similar (full bridging of the defect and histological evidence of new bone growth) to the use of bulk PEA deposited using solvent‐casting.^17^ We found that pPEA was not effective in promoting regeneration if only coated with FN, which reveals the importance of the minimal amount of BMP‐2 used (≈15 ng) (Figure 3b). It is noteworthy that the use of pPEA and pPEA + FN in the mouse model came closer to bridging the bone defect than did the use of PCL alone (Figure 3b), indicating that passive adsorption of adhesion proteins and GFs from the mouse host might also occur onto the scaffold. Nevertheless, our data indicate that engineering adhesion in this way can only enhance osteogenesis to a certain extent. To regenerate bone and repair large defects in a reasonable time, synergistic signalling between integrins and GF receptors is required in vivo.

Further, we translate the technology to demonstrate the application and efficacy of this approach in promoting bone regeneration in a veterinary case of an infected nonunion fracture. In a previously reported recent case study, dogs suffering from nonunion long‐bone fractures were treated with a compression‐resistant matrix soaked in a solution of 0.5 mg mL^−1^ BMP‐2.^40^ Here, we report the successful repair of a nonunion fracture in a dog using decellularized bone chips treated with a novel pPEA + FN + BMP‐2 coating using only 10% of the BMP dose previously reported.^40^ The bone chips themselves facilitated healing of the defect only through osteoconduction, as this kind of graft material has no inherent osteoinductive capacity.^44^ Fracture nonunion in our hospital is uncommon, and these cases are highly variable, so it was not possible to treat a control animal with similar injury, infection, and compromised bone healing. Our conclusions should therefore not be overstated; however, based on clinical experience, we feel that the rapid and robust bone healing observed would not have occurred had the surgery been supplemented using bone chips alone. We note that the treatment of infected critical‐sized defects, to which we demonstrate a regenerative approach, is a major clinical challenge in orthopedic surgery.

Other strategies to present GFs from a material surface, including protein engineering techniques, the use of peptides that bind heparin and GFs,^45^ and the use of layer‐by‐layer technologies,^41^ are reported to be more effective than the soluble administration of GFs.^46^ However, these approaches do not exploit the synergy between integrin and GF receptors to enhance accelerated regeneration.^47^

## 4 Conclusion

To conclude, we show here that the delivery of BMP‐2 at ultralow doses in synergy with integrin‐binding regions of FN can overcome the current obstacles facing orthopedic treatments, such as the regeneration of infected bone. This system has the potential to be developed into a safe, efficient, and cost‐effective therapeutic approach for delivering BMP‐2 at low doses to stimulate bone regeneration and demonstrates the clinical potential of new biomaterials. The study represents a first translation of a polymer/biological interface that targets reproducible molecular control of cell phenotype through multiple cell receptor targeting. This strategy can be potentially used in other applications where the use of growth factors is important to achieve cellular effects, such as cardiovascular tissue engineering, regeneration of osteochondral defects, and nerve repair.

## 5 Experimental Section

*Plasma Polymerization*: Circular 12 mm diameter microscopy cover glasses were sonicated for 25 min in ethanol and treated in air plasma for 5 min before being exposed to monomer plasma. Plasma polymerization of the ethyl acrylate monomer (E9706, Sigma‐Aldrich) onto the substrates was carried out in a custom‐made capacitively coupled plasma installation for low‐pressure plasma in a 15‐L T‐shaped reactor made of borosilicate and stainless steel end plates sealed with Viton O‐rings. Vacuum was produced by a rotary pump or a scroll pump (both BOC Edwards), with operating experiment pressures for the monomer plasma from 0.09 to 0.45 mbar. The plasma was initiated via two capacitively coupled copper band ring electrodes situated outside of the reactor chamber and connected to a radio frequency power supply (Coaxial Power System Ltd.) that works at 13.56 MHz up to 300 W. The monomer pressure was controlled via speedivalves (BOC Edwards) and monitored with a pirani gauge (Kurt J. Lesker). Control samples were prepared by spin‐coating PEA dissolved in toluene at 4% wt/wt. PEA sheets were prepared by radical polymerization of ethyl acrylate solution using 1% benzoin as photoinitiator. A 100 µL droplet of the polymer solution was placed on the glass coverslip, and spin coating was operated at a speed of 3000 rpm and an acceleration of 3000 rpm s^−1^ for 30 s. The PEA‐coated coverslips were then subjected to solvent extraction under vacuum at 60 °C for at least 2 h to ensure that no traces of solvent remained on the surface.

*XPS*: X‐ray photoelectron spectra were obtained at the National EPSRC XPS Users' Service (NEXUS) at Newcastle University, an EPSRC Mid‐Range Facility. XPS was performed using a K‐Alpha apparatus (Thermo Scientific), with a microfocused monochromatic Al Kα source (X‐ray energy = 1486.6 eV) at a voltage of 12 kV, current of 3 mA, power of 36 W, and spot size of 400 µm × 800 µm. Spectra analysis and curve fitting were performed using CasaXPS software version 2.3.16.

*AFM*: Human FN (1918‐FN, R&D Systems) was prepared at 20 µg mL^−1^ in Dulbecco's phosphate‐buffered saline (DPBS) and a 200 µL droplet was placed on the surface of glass coverslips treated with either spin‐coated PEA (SC‐PEA) or pPEA. The protein was allowed to adsorb for 10 min and the remaining liquid was thereafter removed from the surface. The surface was then washed twice with DPBS and once with Milli‐Q water and dried under a stream of nitrogen before AFM imaging. A JPK Nanowizard 4 (JPK Instruments) apparatus was used for imaging in tapping mode using antimony‐doped Si cantilevers with a nominal resonant frequency of 75 kHz (MPP‐21 220, Bruker). The phase signal was set to 0 at a frequency 5–10% lower than the resonant frequency. Height and phase images were acquired from each scan. The JPK Data Processing software version 5 was used for image analysis.

*Protein Adsorption Assays*: ELISA was performed to assess the exposure of specific domains on the FN molecule. After substrates had been coated with 20 µg mL^−1^ FN in DPBS for 1 h, they were blocked for 30 min with 1% bovine serum albumin (BSA, A7979, Sigma‐Aldrich) in DPBS. Next, antibodies for the FN(III_9–10_) domain (HFN7.1, mouse monoclonal, 1:330, Developmental Studies Hybridoma) or FN(III_12–14_) domain (P5F3, sc‐18 827, mouse monoclonal, 1:30, Santa Cruz Biotechnology) were added onto the surfaces and incubated for 1 h. The surfaces were thereafter washed 3 × 5 min with 0.05% Tween 20 in DPBS (PBST). Then, a horseradish peroxidase (HRP)‐conjugated antimouse antibody (626 520, 1:200, ThermoFisher) was added onto the surface and incubated for 1 h in the dark, following by washing for 3 × 5 min with PBST. A substrate solution (DY999, R&D Systems) was then added onto the surfaces and the samples were incubated in the dark for 20 min, followed by the addition of a stop solution (DY994, R&D Systems). The absorbance of the colored solution was read at 450 and 540 nm and the data were used to determine the relative exposure of the FN domains. All procedures were performed at room temperature.

ELISA was also performed to quantify BMP‐2 adsorption. After FN was coated at 20 µg mL^−1^ for 1 h, the surfaces were blocked with 1% BSA in DPBS for 30 min, followed by adsorption of BMP‐2 (355‐BM, R&D Systems) in DPBS for 1 h. The surfaces were washed and blocked again with 1% BSA for 30 min. Next, primary antibodies against BMP‐2 (ab14933, rabbit polyclonal, 1:2000, Abcam) were added onto the surfaces and incubated for 1 h. After washing for 3 × 5 min with PBST, a biotinylated antirabbit antibody (BA‐1100, 1:10 000, Vector Laboratories) was added onto the surfaces and incubated for 1 h. The samples were then washed again for 3 × 5 min with PBST, and a streptavidin‐HRP solution (DY998, R&D Systems) was added and incubated for 20 min. After a final 3 × 5 min wash with PBST, a substrate solution was added onto the surfaces and the samples were incubated in the dark for 20 min, followed by the addition of a stop solution. The absorbance of the colored solution was read at 450 and 540 nm and the data were used to determine the relative adsorption of BMP‐2. All procedures were performed at room temperature.

*Quantification of BMP‐2 Release from pPEA‐Coated Surfaces*: To determine the degree of BMP‐2 release from pPEA‐FN‐coated surfaces, BMP‐2‐loaded samples were incubated at 37 °C for 1 h. At 14 different time points (2 h and 1, 2, 3, 4, 5, 7, 8, 9, 10, 11, 12, 13, and 14 d), supernatant was collected and samples were replenished with fresh buffer. The amount of BMP‐2 in the supernatant was measured using sandwich ELISA (DY355, R&D Systems) following the manufacturer's instructions. Briefly, ELISA plates were coated with the capture antibody and then blocked with BSA for 1 h. After appropriately diluted supernatants were added, bound BMP‐2 was detected with biotinylated antihuman BMP‐2. Streptavidin‐conjugated HRP was then added to the plates. Enzyme substrate (tetramethylbenzidine and peroxide) was treated for 20 min, and the reaction was stopped by adding an acidic solution. Absorbance was measured at 450 nm with wavelength correction at 570 nm. The standard curve was calculated using a four‐parameter logistic (4‐PL) curve fit. The amount of BMP‐2 was calculated from a standard curve based on known concentrations of BMP‐2. Experiments were performed with three replicates of each time point.

*Immunogold Characterization of FN‐GF Interactions*: For immunogold characterization using AFM, immunogold staining was performed on SC‐PEA‐ and pPEA‐modified glass surfaces coated with 3 µg mL^−1^ FN and subsequently with 25 ng mL^−1^ BMP‐2. The samples were washed three times with DPBS and fixed with 4% formaldehyde. They were then incubated with primary antibodies against human BMP‐2 (MAB3551, mouse monoclonal, 1:50, R&D Systems) for 1 h at room temperature. After washing the samples three times with 0.5% Tween‐20 in DPBS (wash buffer), an antimouse immunogold reagent conjugated to 15 nm gold nanoparticles (815.022, 1:50, Aurion) was added to the samples for 1 h at room temperature. The samples were then rinsed with wash buffer and fixed again with 4% formaldehyde and imaged using AFM.

*Cell Culture*: hMSCs (PromoCell) were used for the experiments. Cells were cultured in Dulbecco's Modified Eagle's Medium (D5671, Sigma‐Aldrich) with 10% fetal bovine serum (10 500‐064, ThermoFisher), 0.4% penicillin/streptavidin (P0781, Sigma‐Aldrich), 1 × nonessential amino acids (11 140‐035, ThermoFisher), and an antibiotic mix consisting of 1 × 10^−3^ m sodium pyruvate (S8638, Sigma‐Aldrich), 1 × 10^−3^ m L‐glutamate (G7513, Sigma‐Aldrich), and 0.5% Fungizone (15 290‐018, ThermoFisher). Cells were incubated in a 5% humidified CO_2_ atmosphere at 37°C. Cells were used at passages 2–3 and experiments were performed in triplicates. For all experiments, cells were cultured initially without serum for 3 h.

*Colocalization Studies*: Cells were washed with DPBS and fixed with 4% formaldehyde solution at 4 °C for 15 min. Cells were then permeabilized with a solution of 0.1% Triton X‐100 in DPBS at 4 °C for 10 min. A 1% BSA solution was added and the cells were incubated at room temperature for 30 min to block nonspecific binding. After blocking, primary antibodies (antivinculin, V9131, mouse monoclonal, 1:400, Sigma‐Aldrich; anti‐BMPR1A, PA5‐11 856, rabbit polyclonal, 1:50, ThermoFisher) were added to the cells and incubated at 37 °C for 1 h. Cells were then washed with 0.5% Tween‐20 in PBS (PBST) for 3 × 5 min. Thereafter, AlexaFluor 488 donkey α‐mouse (A21202, 1:200, ThermoFisher) and Cy3‐conjugated goat α‐rabbit (711‐165‐152, 1:100, Jackson ImmunoResearch) were added to the cells and incubated at 37 °C for 1 h, followed by PBST washing for 3 × 5 min. The nuclei of the cells were stained using VectaShield‐4′,6‐diamidino‐2‐phenylindole (DAPI) (H‐1200, Vector Laboratories), while samples were mounted on glass slides for fluorescence microscopy.

*Western Blot*: Cell lysates were harvested after culturing on various surfaces for 1 h, using radioimmunoprecipitation (RIPA) buffer supplemented with protease inhibitors (S8830, Sigma‐Aldrich) and phosphatase inhibitors (78 427, ThermoFisher). Western blotting was performed using the same amount of protein in each sample in denaturing conditions for pSMAD 1/5/9 (12656T, rabbit monoclonal, 1:1000, Cell Signaling) and pFAK (05‐1140, mouse monoclonal, 1:500, EMD Millipore). After membranes were washed in Tris‐buffered saline with Tween 20 for 6 × 5 min, antirabbit (7074, 1:2000, Cell Signaling) and antimouse (NA931VS, 1:10 000, Abcam) secondary antibodies were added, and a chemiluminescent HRP substrate for immunodetection (Millipore, WBKLS0500) was used before X‐ray detection. Protein expression was quantified using ImageJ and normalized with total protein amount measured by BCA (23 225, ThermoFisher).

*ALP Assay*: Cells grown on coverslips were rinsed twice with DPBS and harvested prior to ALP assay. Each coverslip with attached cells was broken into pieces and transferred to a 1.5 mL Eppendorf tube. Then, 500 µL of ice‐cold Tris‐HCl was added to each tube. The sample was sonicated twice for 10 s each at 5 W and centrifuged at 10 000 × g for 5 min. The supernatant was transferred to a new tube and the protein concentration of each sample was measured by BCA assay (23 225, ThermoFisher). Then, a sample volume corresponding to 25 µg of protein (adjusted to 25 µL with DPBS) was added to each well of a black 96 well plate, and 100 µL of a fluorescent 4‐methylumbellifeyl phosphate disodium salt (MUP, M8168, Sigma‐Aldrich) substrate solution, made by mixing 500 µL of 1 m NaHCO_3_, 2 mL of 50 × 10^−3^ m diethanolamine, 7.5 mL of deionized water, and 34 µL of 25 mg mL^−1^ MUP stock, was added to each well. After incubating at 37 °C for 1 h in the dark, the fluorescence of the plate was read at an excitation wavelength of 360 nm and an emission wavelength of 465 nm. ALP standards were prepared using serial dilutions of a 10 mU µL^−1^ ALP solution (P6774, Sigma‐Aldrich) to obtain a calibration curve.

*Immunofluorescence of Osteogenesis‐Related Markers*: Cells were washed with DPBS and fixed with 4% formaldehyde solution at 4 °C for 15 min. Cells were then permeabilized with a solution of 0.1% Triton X‐100 in DPBS at 4 °C for 10 min. Blocking was performed using 1% BSA at room temperature for 30 min. Primary antibodies (anti‐OPN, sc‐21 742, mouse monoclonal, 1:100, Santa‐Cruz Biotechnology; anti‐OCN, sc‐73 464, mouse monoclonal, 1:100, Santa‐Cruz Biotechnology) were added to the cells and incubated at 37 °C for 1 h. Cells were then washed with PBST for 3 × 5 min. Thereafter, AlexaFluor 488 donkey α‐mouse (A21202, 1:200, ThermoFisher) and Cy3‐conjugated goat α‐rabbit (711‐165‐152, 1:100, Jackson ImmunoResearch) were added to the cells and incubated at 37 °C for 1 h in the dark, followed by PBST washing for 3 × 5 min. The nuclei of the cells were stained using VectaShield‐DAPI, while samples were mounted on glass slides for fluorescence microscopy.

*Implant Preparation for In Vivo Study of Nonunion Critical‐Sized Bone Defect*: Thin, porous polyimide sleeves (Microlumen) were coated by solvent casting of a PCL solution, creating a polymer layer on the walls of the tube, followed by the deposition of a thin layer (<1 µm) of pPEA to cover the underlying PCL polymer. Then, FN or FN + BMP‐2 was adsorbed on the cylindrical polymer surface. Implant tubes without BMP‐2 and tubes coated with the sole polymer layer of PCL or PCL + pPEA were used as controls. Implant tubes covered with solvent‐casted PEA coated with FN and BMP‐2 were used as a positive control, this condition being the one that showed a higher potential in promoting bone regeneration in the preliminary experiment.^17^ At least three replicates per group were used in the experiment. It was important to note that implant tubes coated was used with the polymer of interest instead of 3D scaffolds, due to the size limitations of the small animal model.

*Nonhealing Bone Defect Model in Mice*: All in vivo experiments were conducted under the Animals (Scientific Procedures) ACT 1986 (ASPel project license n° 70/8638). Male B6‐129 mice (8–10 weeks old, Charles River) were used for these studies. Mice were fully anesthetized using isoflurane gas as an anesthetic agent. To create bone defects, the midsection of the radius and ulna in the right front paw of the mouse was exposed. A custom‐built double‐blade bone cutter was used to precisely generate a 2.5 mm segmental defect on the radius without disturbing the ulna. The implant tube (4.0‐mm long) was fitted over the ends of the defect. After repositioning the muscle and skin, the wound was closed and the mouse was completely ambulatory following recovery. Figure S11 in the Supporting Information shows a scheme of the segmental bone model used.

*Analysis of New Bone Formation*: Bone growth in the area of the defect was evaluated 8 weeks after implantation. Bone samples were explanted and analyzed by X‐ray (SARRP, Perkin Elmer 0820 detector panel) and µCT scanning (Bruker Skyscan Micro X‐ray CT). Then, tissue samples were decalcified and embedded in paraffin and histological sections were stained with hematoxylin‐Safranin O‐fast green. 3D reconstructions of 4 mm length of the bone radio in the area of the defect were obtained by contouring 2D slices from the µCT scans. Quantification of the volume and specific surface of new bone within the defect was performed using the free CTAn software from Bruker. In order to ensure that only new bone formation was measured, the volume of interest (VOI) was selected to evaluate a central 2.0 mm length in the area of the defect. The 3D volume measurement was based on the marching cubes volume model of the binarized objects within the VOI. The specific surface was evaluated as the ratio of solid surface to volume measured in 3D within the VOI. This was a basic parameter to assess the thickness and complexity of structures, and thus was useful to characterize the complex porous structure of the bone.

*Preparation of Bone Chips for Implantation*: Commercially available dog bone chips (Veterinary Instrumentation Ltd.) were coated with pPEA. A total of 5 cc of cancellous canine chips, with a chip size of 2–4 mm, were spread on a glass petri dish and placed in the aforementioned custom‐made plasma chamber. The chips were coated with a plasma power of 100 W for 30 min. Monomer pressure during plasma was 1.8 to 2.4 × 10^−1^ mbar. After plasma, the chips were sterilized under UV light for 15 min and then coated with adsorbed FN and BMP‐2. First, 10 mL of FN in DPBS at 20 µg mL^−1^ was used to coat the chips, using vacuum to ensure that the liquid reached the entire surface of the chips. After 1 h of adsorption, the remaining liquid was removed using a pipette. Then, 6 mL of BMP‐2 at 50 µg mL^−1^ was added to the chips. Vacuum was once again used to ensure full surface coating. The chips were moved into the operating theater and after 2 h of GF adsorption, the chips were spread on a surgery gauze to soak all the remaining liquid and thereafter directly used by the veterinary surgeon.

*Clinical Veterinary Case— Details of Surgery and Postoperative Care*: The local ethics committee was consulted regarding this novel procedure, and the informed consent of the dog's owner was obtained prior to the procedure. The Münsterländer dog (2‐year‐old female, neutered, body weight 21 kg) was anesthetized using a standard protocol: premedication with methadone (6 mg IM) and medetomidine (100 µg IM), induction with propofol (30 mg IV), and maintenance with isoflurane in oxygen. The entire right humerus and proximal third of the left humerus were clipped and aseptically prepared for surgery. For analgesia, a brachial plexus nerve block was performed preoperatively using 20 mg of levobupivacaine, and methadone (6 mg IV) was given once during surgery. A standard surgical approach was made to the fractured humerus, on its medial aspect. Approximately 1 cm of the nonhealing ends of the bone were excised, along with all soft tissue within the fracture gap. The fracture was stabilized using a 3.5 mm locking compression plate (Synthes) placed medially and a 2.7‐mm locking compression plate (Synthes) placed cranio‐medially. A standard surgical approach was made over the greater trochanter of the left humerus and a hole was drilled in the lateral cortex using a 4.5 mm drill bit. A curette was used to harvest cancellous bone from the humeral head, which was then mixed with 5 cc of pPEA‐chips (decellularized bone chips coated with pPEA using plasma polymerization and subsequently with FN and BMP‐2, as previously described). The combined graft materials were placed within the fracture gap using Debakey forceps. The muscle layer was closed using polydioxanone in a simple continuous pattern and skin was closed using poliglecaprone 25 in an intradermal pattern. Surgical time was 4 h and 5 min and recovery from anesthesia was rapid and smooth. For postoperative analgesia, methadone (6 mg IM) was given every 4 h for the first day postoperatively and meloxicam (2 mg PO SID) was prescribed for 2 weeks. Seven weeks after surgery, the dog was sedated with medetomidine (200 µg IV) and butorphanol (2 mg IV) for radiography of the humerus, and recovered well from this procedure. The dog attended twice weekly physiotherapy sessions for 5 months postoperatively, and daily physiotherapy exercises were encouraged at home. For the first 7 weeks, physiotherapy included the application of pulsed electromagnetic field therapy to the fracture site (50 Hz, constant pulse, for 30 min twice daily, Biomag 2 Therapy Unit, Westville Therapy). The dog's exercise was restricted to short controlled lead walks for around 3 months after surgery; it was then progressively increased as limb function improved.

*Statistical Analysis*: Experiments were performed in triplicates (*n* = 3) and data were expressed as mean ± SD. Statistical analysis was performed by one‐way analysis of variance (ANOVA) with Tukey's test for multiple comparisons using OriginPro 8. Statistical significance was defined as *p* < 0.05. For in vivo experiments, data were presented as mean ± SD, minimum *n* = 3. Two‐tailed t‐test was used to analyze data, *p* < 0.1.

## Conflict of Interest

The authors declare no conflict of interest.

## Supporting information

Supplementary

Supplementary
