# Supplementary material for: Nanoscale Coatings for Ultralow Dose BMP‐2‐Driven Regeneration of Critical‐Sized Bone Defects
Source: Adv Sci (Weinh). 2018 Nov 19;6(2):1800361. doi: 10.1002/advs.201800361 (PMC6343071; doi:10.1002/advs.201800361)
Supplement: Supplementary file 1 — Supplementary [file ADVS-6-1800361-s002.pdf]

Copyright WILEY-VCH Verlag GmbH & Co. KGaA, 69469 Weinheim, Germany, 2018.

ADVANCED SCIENCE
Open Access

Supporting Information

for *Adv. Sci.,* DOI: 10.1002/advs.201800361

Nanoscale Coatings for Ultralow Dose BMP-2-Driven Regeneration of Critical-Sized Bone Defects

Zhe A. Cheng, Andres Alba-Perez, Cristina Gonzalez-Garcia, Hannah Donnelly, Virginia Llopis-Hernandez, Vineetha Jayawarna, Peter Childs, David W. Shields, Marco Cantini, Laura Ruiz-Cantu, Andrew Reid, James F. C. Windmill, Elena S. Addison, Sandra Corr, William G. Marshall, Matthew J. Dalby, and Manuel Salmeron-Sanchez**

Copyright WILEY-VCH Verlag GmbH & Co. KGaA, 69469 Weinheim, Germany, 2018.

Supporting Information

Nanoscale coatings for ultra-low dose BMP-2-driven regeneration of critical-sized bone defects

Zhe A. Cheng, Andres Alba-Perez, Cristina Gonzalez-Garcia, Hannah Donnelly, Virginia Llopis-Hernandez, Vineetha Jayawarna, Peter Childs, David W. Shields, Marco Cantini, Laura Ruiz-Cantu, Andrew Reid, James F. C. Windmill, Elena S. Addison, Sandra Corr, William G. Marshall, Matthew J. Dalby, and Manuel Salmeron-Sanchez**

Supplementary Materials and Methods

Scratch test for polymer coating thickness: After plasma polymerisation of poly(ethyl acrylate) (PEA) on glass substrates, a thin scratch was manually made on the surface using a razor blade to expose the underlying substrate. The surface was then viewed under a microscope to identify the scratch. Atomic force microscopy (AFM) was performed across the width of the scratch at a scan rate of 0.3 Hz. The difference in height profile between the scratched and unscratched area is taken to be the thickness of the polymer layer.

Pull-off test for PEA adhesion: Glass coverslips were coated with pPEA for 30 minutes at 100 W. The bonding strength was determined by the pull-off test (DIN ISO 4624:2016) using the Zwick/Roell Z2.0 testing machine. Test dollies consisted of an aluminium cylindrical-faced testing body, specifically designed to be used with the tensile tester. Each dolly had a rigid, flat face for bonding the adhesive/coating at one end and a facility for connecting the pull-off tester at the other. Each dolly had a nominal diameter of 12 mm and the faces of each dolly were machined perpendicular to its axis before use. The substrates were glued to the dollies using two-component epoxide glue as an adhesive with 20 of curing, which was the minimum quantity of adhesive required to produce a firm, continuous, and even bond between the components of the test assembly. The test assembly

was aligned in the centering device. Tensile stress was applied at 1 mm/s, perpendicular to the plane of the coated substrate.

Fibronectin (FN) availability: Enzyme-linked immunosorbent assay was performed to assess the availability of the FN molecule on each surface. After substrates have been coated with 20 µg/mL FN in Dulbecco's phosphate-buffered saline (DPBS) for 1 h, they were blocked for 30 min with 1% bovine serum albumin (BSA, Sigma-Aldrich) in DPBS. Next, antibodies for FN (F3648, rabbit polyclonal, 1:10000, Sigma-Aldrich) were added onto the surfaces and incubated for 1 h. After washing for 3 × 5 min with 0.5% Tween in DPBS (PBST), a biotinylated anti-rabbit antibody (BA-1100, 1:10000, Vector Laboratories) was added onto the surfaces and incubated for 1 h. The samples were then washed again for 3 × 5 min with PBST, and a streptavidin-HRP solution (R&D Systems) was added and incubated for 20 min. After a final 3 × 5 min wash with PBST, a substrate solution (R&D Systems) was added onto the surfaces and the samples were incubated in the dark for 20 min, followed by the addition of a stop solution (R&D Systems). The absorbance of the coloured solution was read at 450 nm and 540 nm immediately and the data were used to determine the relative availability of FN on each surface. All procedures were performed at room temperature.

Alizarin red staining: Human mesenchymal stem cells (hMSCs) maintained in culture for 3 weeks were washed with PBS and fixed with 10% formaldehyde for 15 min. Following this, cells were washed with sterile water and incubated with alizarin red solution (40 mM, pH 4.2) for 1 h at room temperature. Cells were then washed until clear with sterile water and stored in sterile water for microscopy.

Quantitative real-time (q)PCR: hMSCs were cultured on scaffolds for 7 days at a density of 1 × 10^4 cells/scaffold. Total RNA was extracted using a Qiagen RNeasy micro kit and the quantity and integrity of the RNA were measured with NanoDrop (ThermoFisher Scientific). Amplification by qRT-PCR was done using human specific primers for BMPR-2, RUNX2, and osterix detailed in Table 1 (all Eurofins Genomics). PCR was carried out using a

7500 real-time PCR system & software (Applied Biosystems). Samples had a total reaction volume of 20 µL containing 2 µL of diluted cDNA, each reverse and forward primer at a final concentration of 100 µM and analysed using SYBR green chemistry (Qiagen). For PCR amplification, samples were held at 50°C for 2 min, then 95°C for 10 min, then amplified at 95°C for 15 s and 60°C for 1 min for 40 cycles. The specificity of the PCR amplification was checked with a heat dissociation curve (measured between 60–95°C) done subsequent to the final PCR cycle. Gene expression levels were standardised using GAPDH as an internal control. Quantification analysis was performed using the comparative ΔΔCt method and gene expression was expressed as fold change relative to the control sample. Samples were assayed in triplicates and gene expression was expressed as mean ± SEM.

Table 1. qPCR primer sequences for SYBR green.

PRIMER	FORWARD	REVERSE
**GAPDH**	TCAAGGCTGAGAACGGGAA	TGGGTGGCAGTGATGGCA
**RUNX2**	GGTCAGATGCAGGCGGCCC	TACGTGTGGTAGCGCGTGGC
**OSX**	GCTTATCCAGCCCCCTTTAC	CACTGGGCAGACAGTCAGAA
**BMPR-2**	GATGGCAAATCAGGATCAGG	CTTCACAGTCCAGCGATTCA

In cell western for pRUNX2: Cells maintained in culture for 5 days were washed with PBS and fixed with 10% formaldehyde for 15 min. Permeabilisation buffer (10.3 g of sucrose, 0.292 g of NaCl, 0.06 g of $MgCl_2$, 0.476 g of HEPES, and 0.5 mL of Triton X in 100 mL of H_2O, at pH 7.2) was added for 4 min at 4°C. Following this, 1% milk protein/PBS was added to block non-specific binding for 1.5 h on a shaker at 37°C, followed by 2 × 5 min washes in 1% milk protein/PBS. Primary antibodies for pRUNX2 (ThermoFisher) were added at 1:50 dilution in 1% milk protein/PBS. Cell Tag 700 (LiCor) was added for 2 h at room temperature to normalise cell number at 1:500 dilution. Subsequent 5 × 5 min washes in wash buffer (PBS

+ 0.1% Tween 20) were performed. The wash buffer was removed and secondary antibodies were added for 1 h in a shaker covered in foil, corresponding secondary antibody (LiCor) diluted 1:10000. The plate was washed 5 × 5 min with wash buffer at room temperature with shaking. The plate was washed once more with PBS and dried before imaging on LiCor Odyssey Sa scanner. Samples were assayed in quadruplicate and total protein was expressed as mean ± SEM, with measured background fluorescence subtracted, *** $p < 0.0005$.

Von Kossa staining: Von Kossa staining was carried out to assess mineralisation. Cells were covered with silver nitrate (5% in deionised water) and exposed to strong UV light for 30 min. Then, the cells were rinsed with deionised water. Sodium thiosulfate (5% in deionised water) was added to the cells and incubated for 10 min. The cells were then rinsed under tepid running tap water for 10 min and rinsed again with deionised water. Counterstaining was performed with nuclear fast red for 10 min, and cells were rinsed with deionised water followed by 70% ethanol.

Preparation of 3D PCL scaffolds: Circular scaffolds were designed with BioCAD software (Regenhu). Overall dimensions were diameter = 5 mm and height = 0.8 mm. The distance between the centres of parallel printed strands was set to 500 μm and the height increase of the printhead between layers was set to 175 μm. Scaffolds were 3D-printed on a REGENHU 3D Discovery bioprinter in a custom-built enclosure with an air temperature of 27°C and 35% relative humidity. Polycaprolactone (PCL) pellets (Mn 80000, Corbion) were extruded at a temperature of 74°C using a nozzle with 300 μm diameter. The printhead travelled at a rate of 16 mms^{-1} and the pressure of the PCL melt chamber was 2 bar.

Scanning electron microscopy (SEM): SEM of 3D PCL scaffolds was performed using an FEI Nova NanoSEM 630 in secondary electron mode at a voltage of 5 kV and a working distance of approximately 7 mm. Prior to SEM imaging, the scaffolds were coated with Au/Pd 80/20 to avoid charging effects.

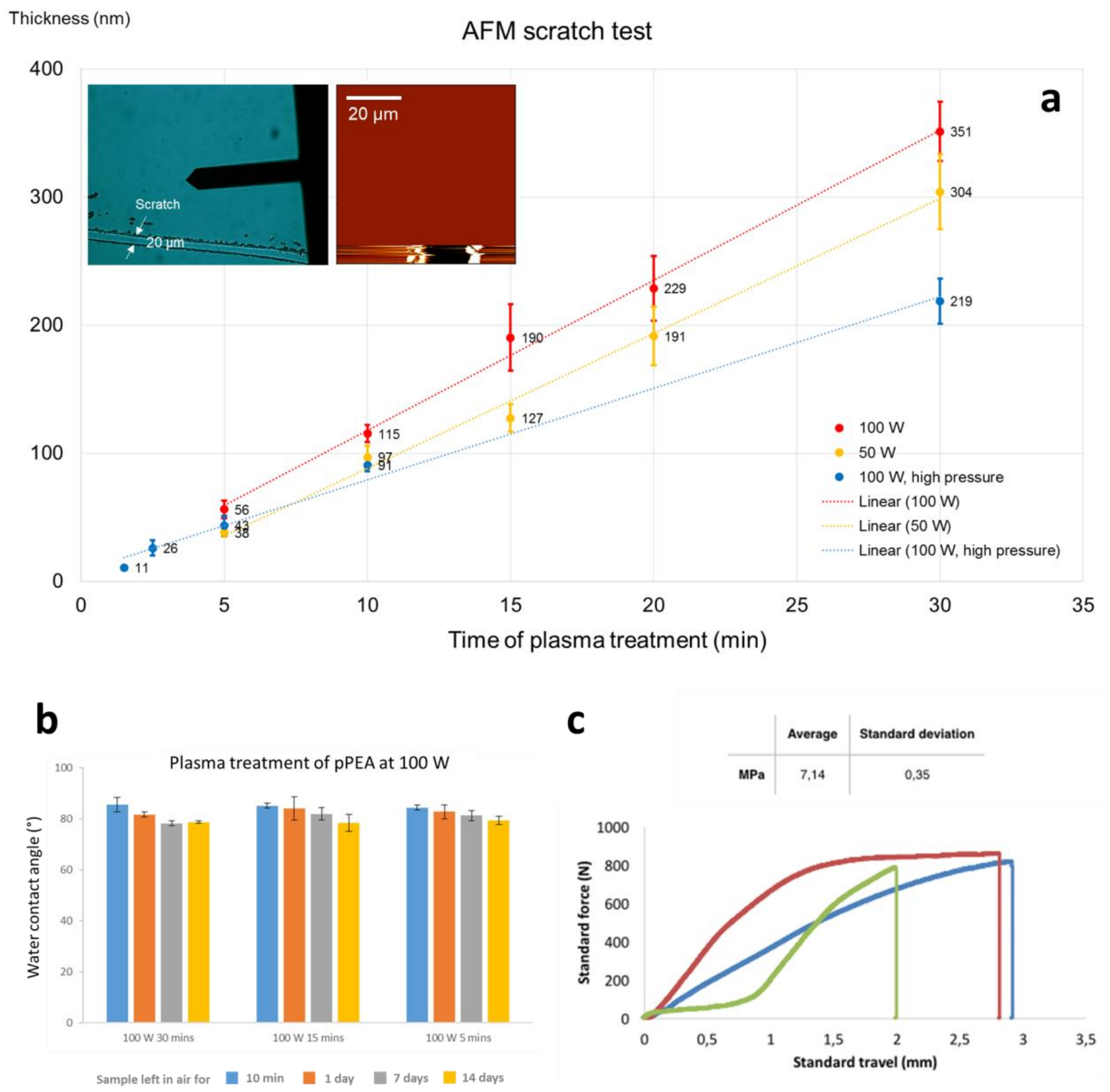

Figure S1. Characterisation of the pPEA coatings deposited at various plasma conditions. **(a)** Thickness of pPEA coatings measured with a scratch test using atomic force microscopy (AFM), n = 3 (minimum). Data are presented as mean ± SD. Inset: Microscopic photograph showing AFM cantilever and area of scratch on pPEA-coated surface (left) and partial AFM scan of scratched area (right). **(b)** Water contact angle of pPEA coatings deposited at a plasma power of 100 W, measured after leaving samples in air for various durations, n = 3 (minimum). Data are presented as mean ± SD. **(c)** Standard force (N) curves obtained for each

sample tested (plasma power at 100 W, 30 min of coating). Average and standard deviation of the adhesion force for the pPEA-coated samples.

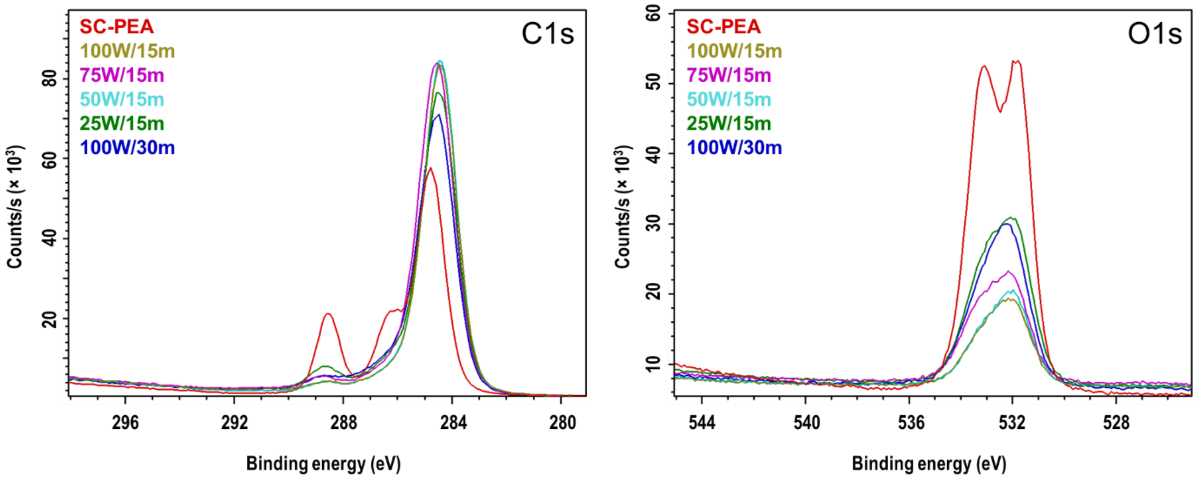

Figure S2. High-resolution XPS of pPEA-coated surfaces with plasma polymerisation performed at different powers for various durations. SC-PEA was used as the control.

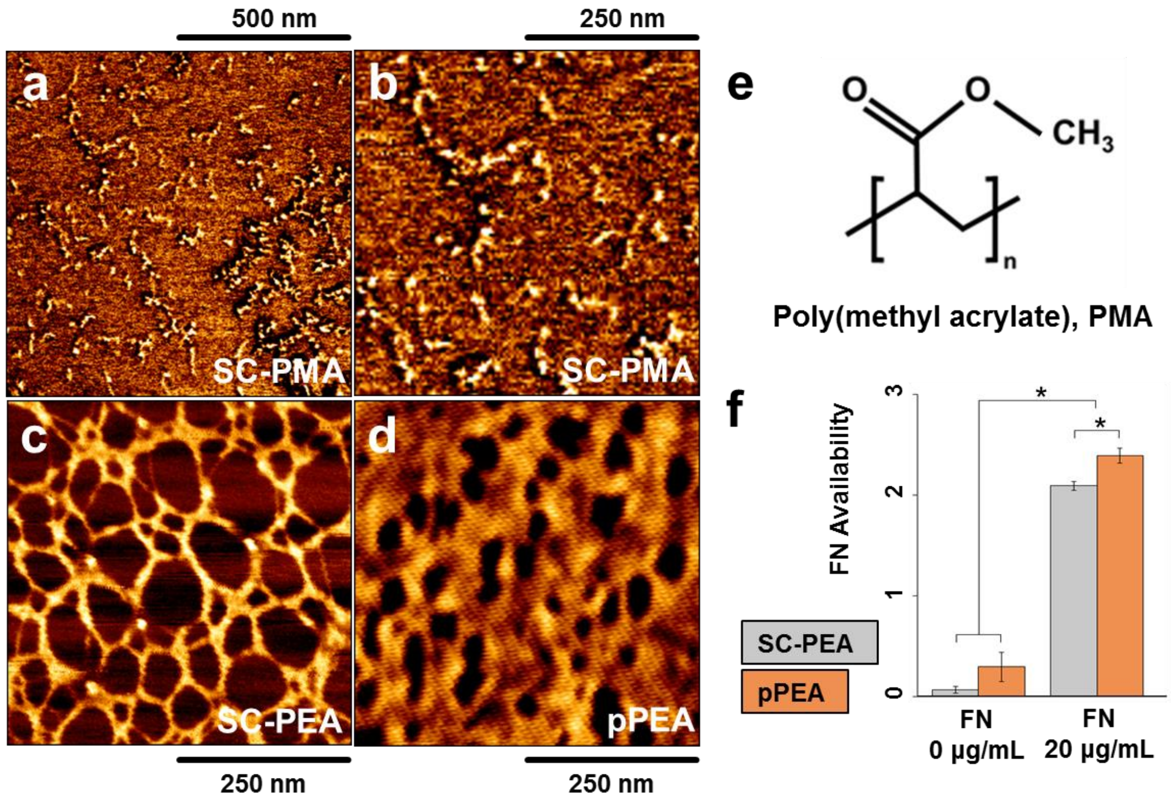

Figure S3. Atomic force microscopy (AFM) phase images of 20 μg/mL fibronectin (FN) adsorbed for 10 min on **(A)** spin-coated poly(methyl acrylate) (SC-PMA), large scan area, **(B)** SC-PMA, small scan area, **(C)** SC-PEA, small scan area, and **(D)** pPEA, small scan area. PMA does not induce fibrillogenesis while both SC-PEA and pPEA do, although the FN networks formed on pPEA are thicker and denser than those on SC-PEA. **(E)** Chemical structure of PMA, which has one fewer carbon atom than PEA. **(F)** Availability of total FN adsorbed at different FN concentrations on SC-PEA and pPEA for 1 h. Data are presented as mean ± SD, n = 3, one-way ANOVA with Tukey's test for multiple comparisons. $^*p < 0.05$.

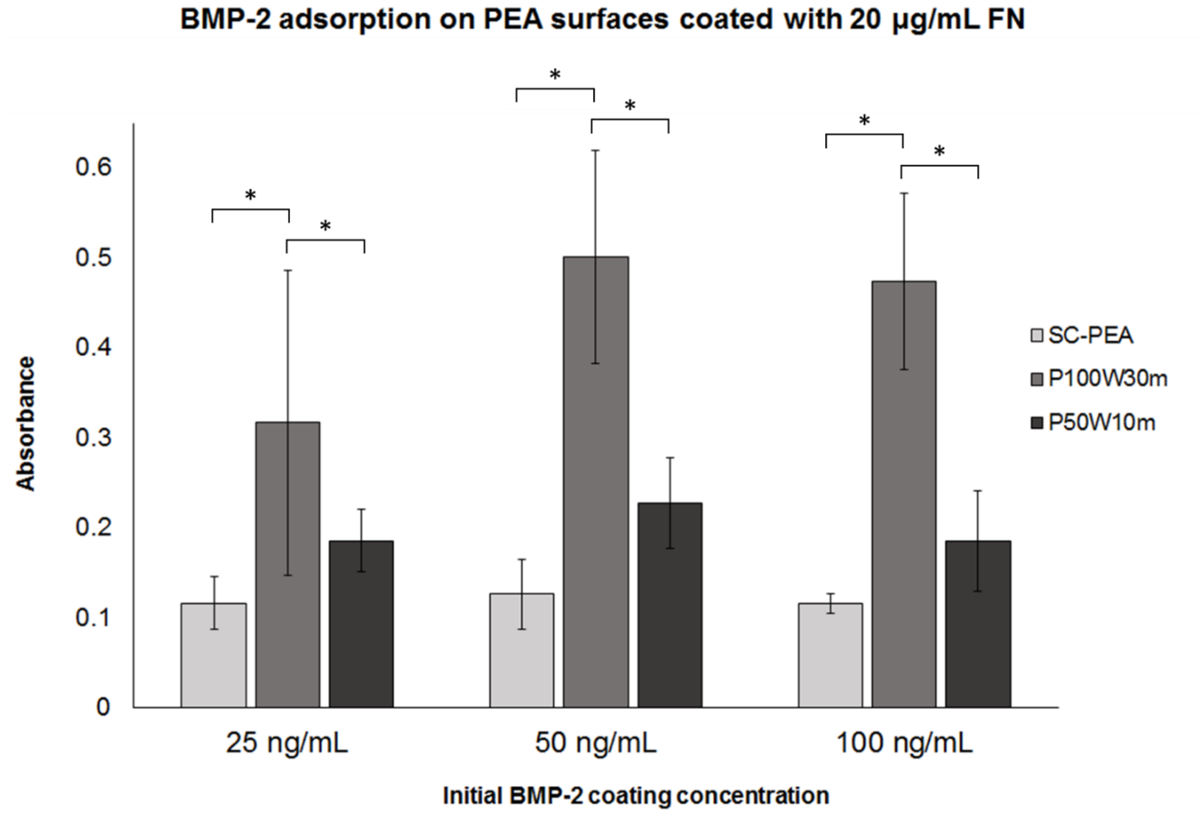

Figure S4. Relative adsorption of different concentrations of BMP-2 on FN-coated surfaces. SC-PEA: spin-coated PEA, P100W30m: pPEA deposited at 100 W for 30 min, P50W10m: pPEA deposited at 50 W for 10 min. Data are presented as mean ± SD, n = 3, one-way ANOVA with Tukey's test for multiple comparisons. *$p < 0.05$.

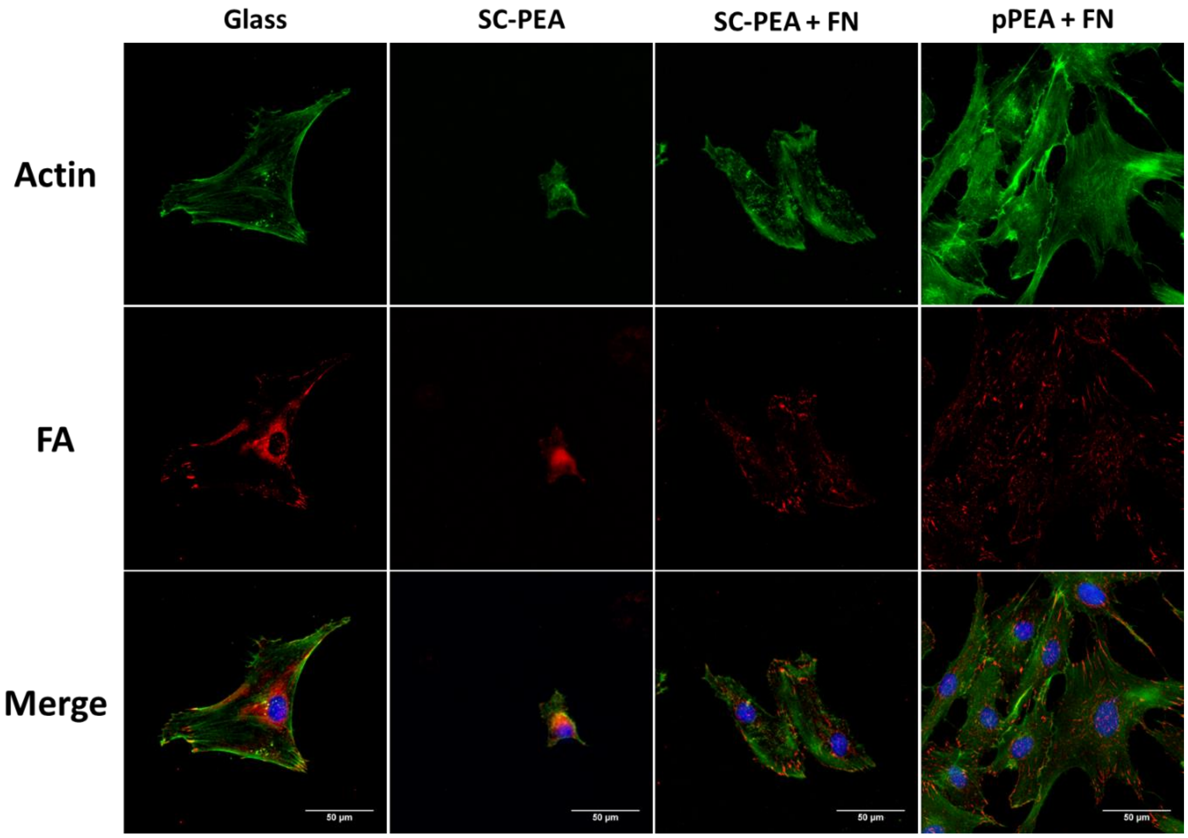

Figure S5. Immunostaining of hMSCs on various surfaces after 24 h in culture. Actin (Phalloidin) is shown in green, focal adhesions (FA) are shown in red, and nuclei are shown in blue in the merged images. Attachment and spreading of hMSCs after 24 h in culture were significantly enhanced on pPEA + FN compared with those on SC-PEA + FN, as evidenced by the larger number of cells, greater degree of spreading, and enhanced focal adhesion formation. Scale bar = 50 µm.

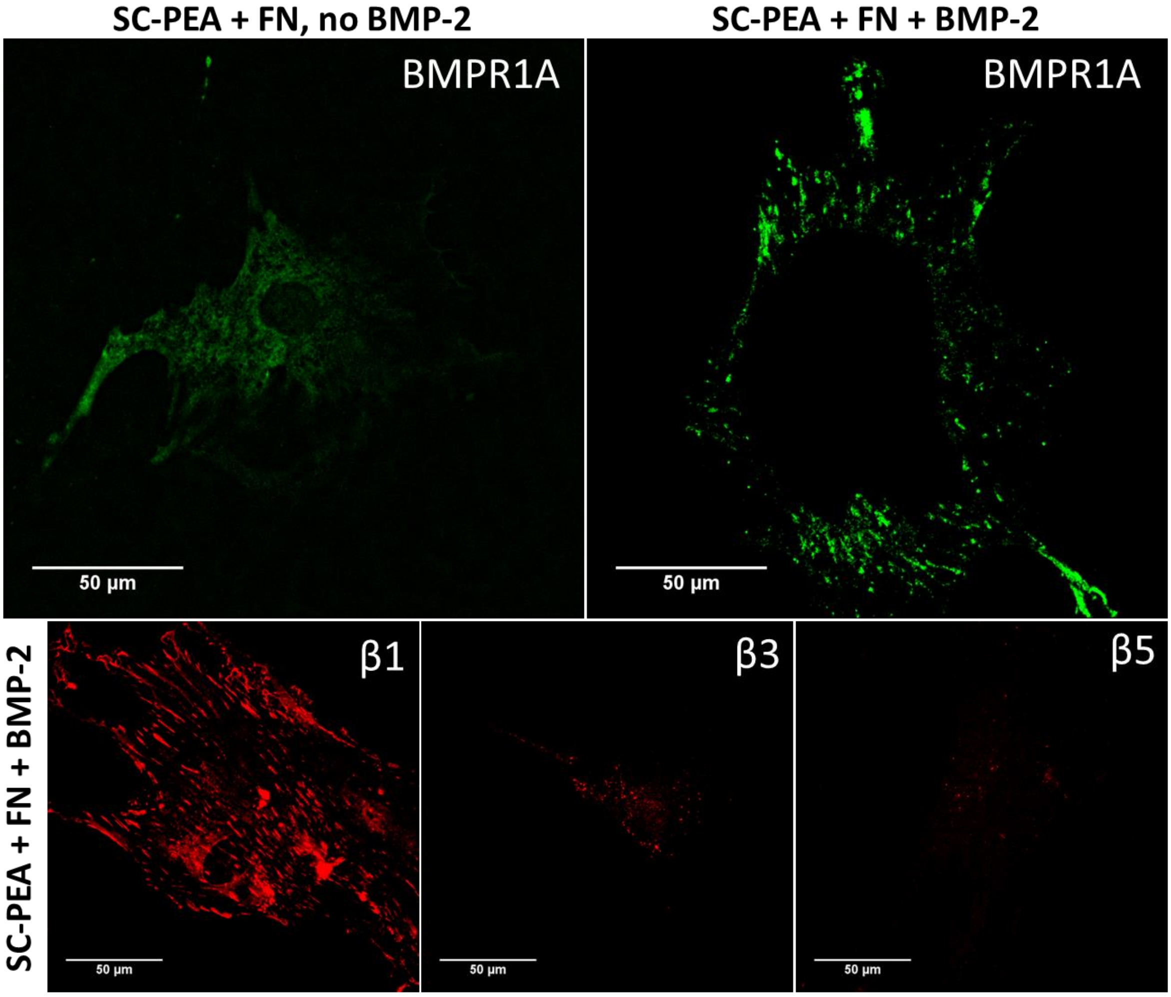

Figure S6. Immunostaining of BMPR1A (top row) in human (h)MSC cultured on surface coated with SC-PEA + FN, without BMP-2 (left) and with 50 ng/mL BMP-2 (right). Positive staining (in green) is evident when BMP-2 is present on the surface and is lacking when BMP-2 is absent. Staining of individual integrins (β1, β3, β5) on surfaces coated with SC-PEA + FN + BMP-2 is shown in the bottom row. β1 is abundantly expressed (in red) while the other integrins are not expressed, showing the specificity of integrin engagement. Scale bar = 50 µm.

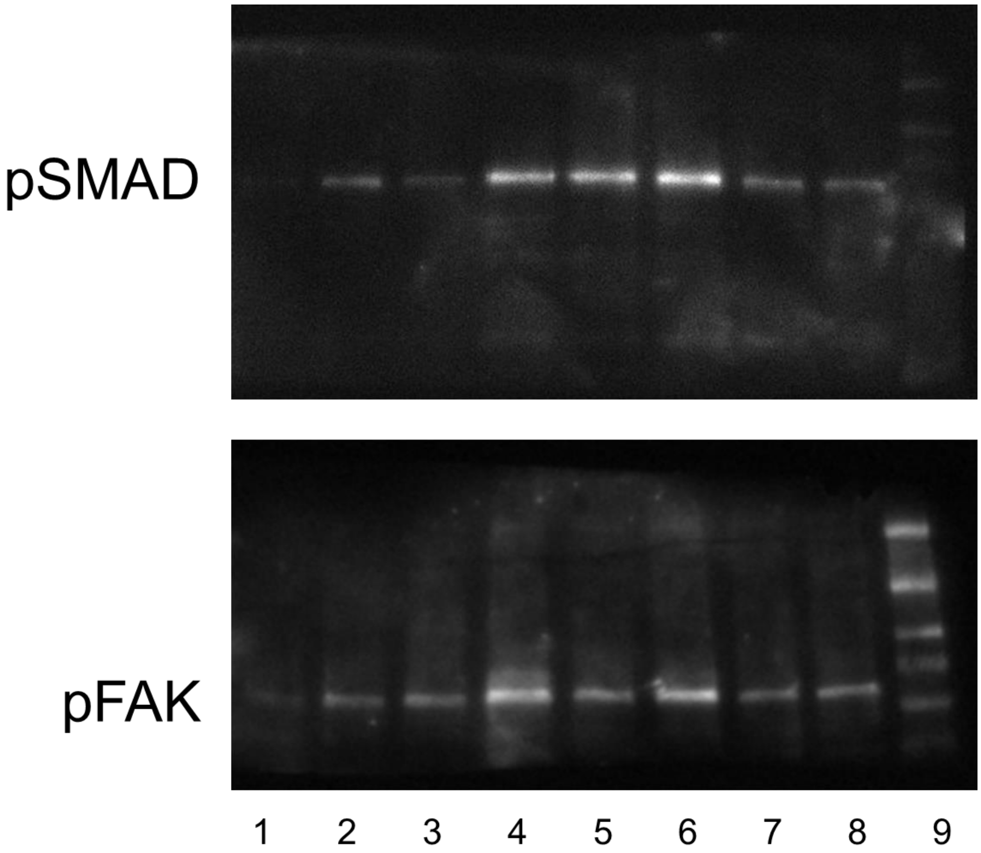

Figure S7. Western blots for pSMAD 1/5/9 and pFAK, expressed by hMSCs after 1 h in culture on (1) bare glass substrate, (2) bare glass substrate in medium with soluble BMP-2, (3) SC-PEA + FN, (4) SC-PEA + FN + BMP-2, (5) pPEA + FN, (6) pPEA + FN + BMP-2, (7) pPEA (low power, short time) + FN, and (8) pPEA (low power, short time) + FN + BMP-2. (9) indicates the size marker.

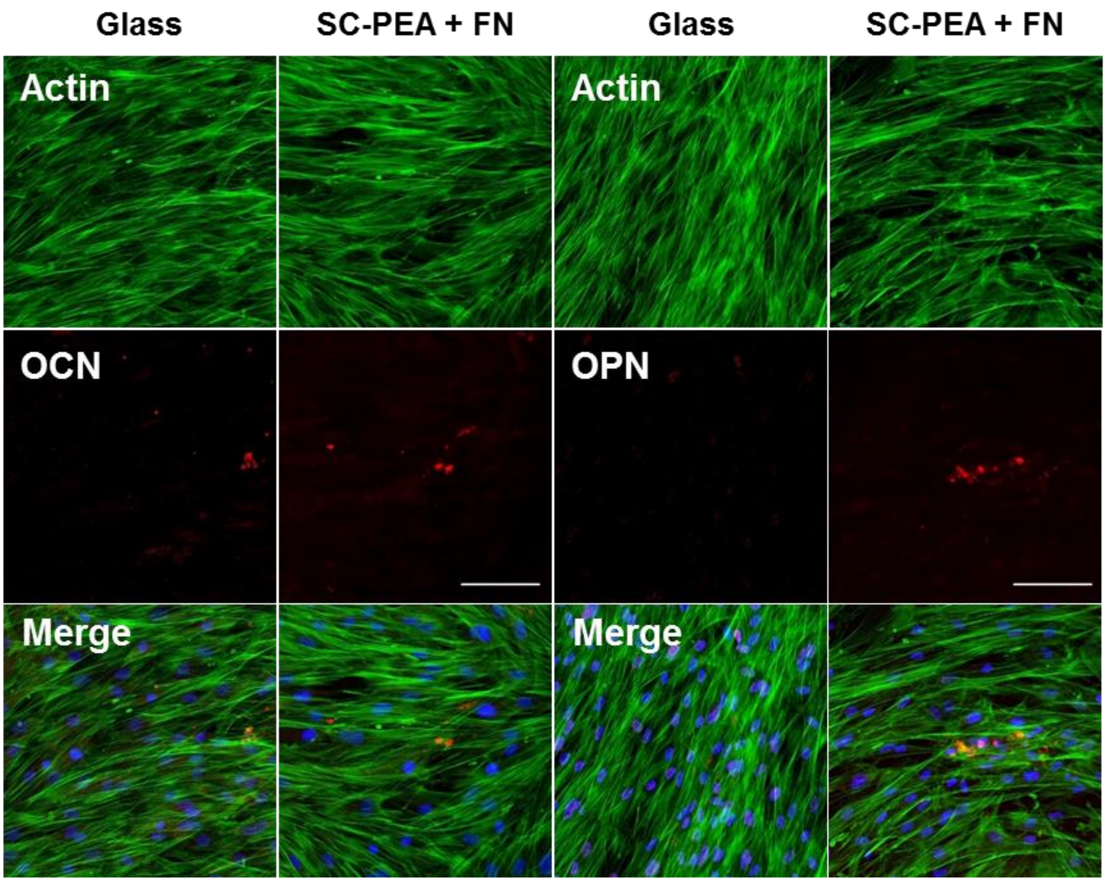

Figure S8. Immunostaining of osteocalcin (OCN) and osteopontin (OPN) in red, actin (Phalloidin) in green, and nuclei in blue in hMSCs cultured on control surfaces (glass and SC-PEA + FN) for 21 days. Scale bar = 50 μm.

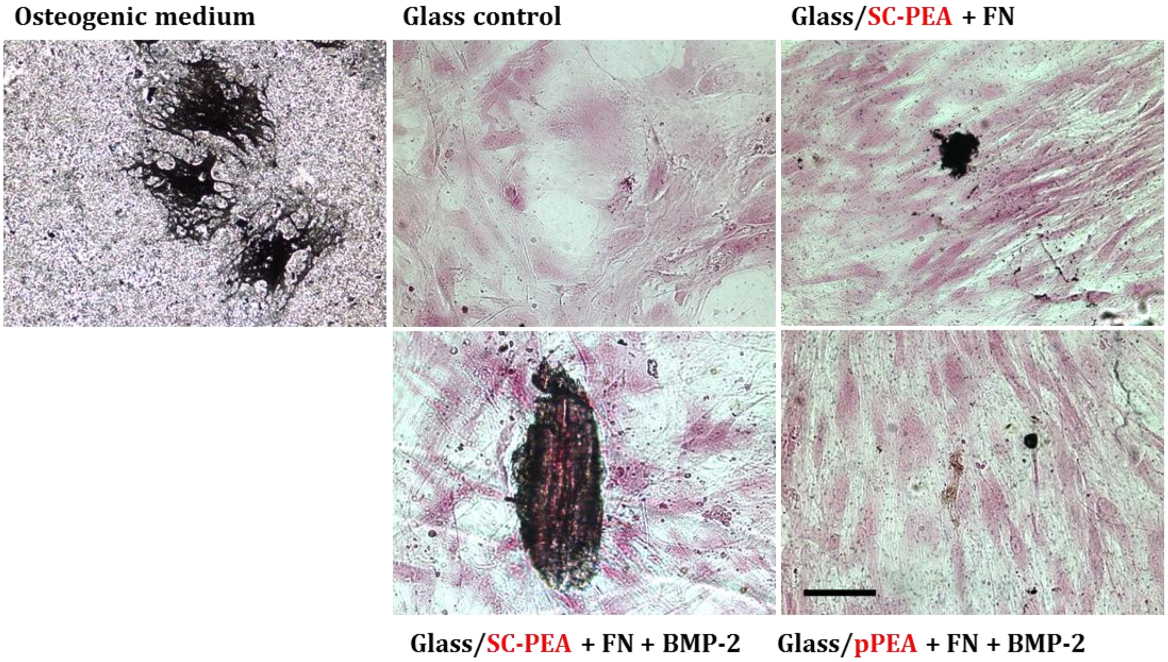

Figure S9. Von Kossa staining of mineralisation in hMSCs cultured on SC-PEA and pPEA surfaces with FN, and with or without BMP-2, for 28 days. Scale bar = 50 µm.

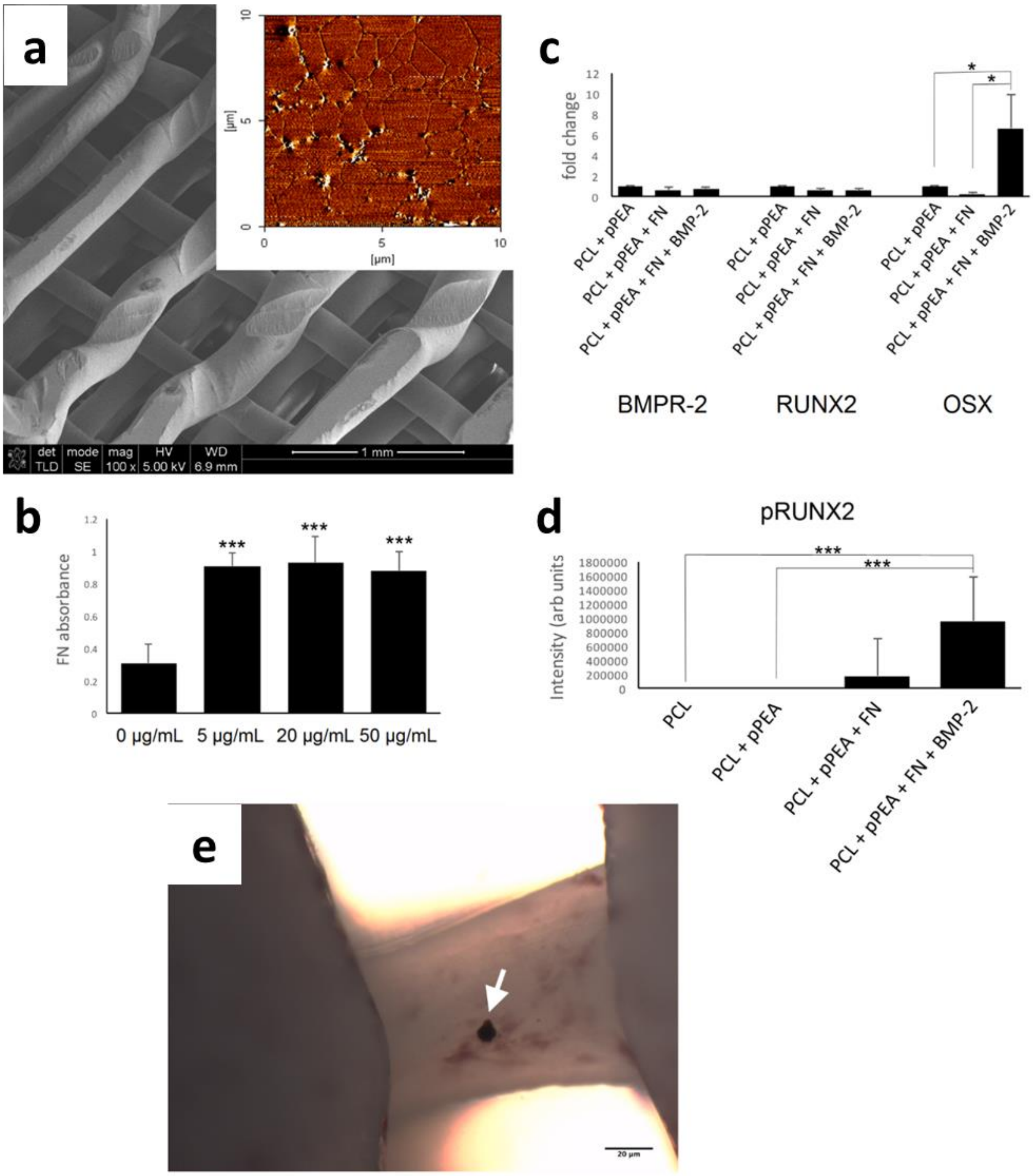

Figure S10. *In vitro* evaluation of pPEA-coated 3D polycaprolactone (PCL) scaffolds. **(a)** Scanning electron micrograph of PCL scaffolds (5 mm diameter, 800 μm high), fabricated by 3D printing (filaments with 200 μm diameter, 500 μm separation, layered at 90° to the last layer). Inset in (A): FN could be seen to form fibrillar nanonetworks on the PCL scaffolds in response to pPEA coating. **(b)** Quantification of FN on pPEA-coated PCL scaffolds. FN was

adsorbed at 20 µg/mL and coated for 30 min. n = 3, results = mean ± SD, *** $p < 0.001$ by one-way ANOVA. **(c)** qPCR showed increased OSX expression from hMSCs cultured on the scaffold coated with pPEA + FN + BMP-2 after 7 days of culture. n = 3, results = mean ± SD, * $p < 0.05$ by one-way ANOVA. **(d)** RUNX2 phosphorylation, evaluated by qPCR, increased at day 5 of culture in response to scaffolds coated with pPEA + FN + BMP-2. n = 4, results = mean ± SD, *** $p < 0.001$ by one-way ANOVA. **(e)** Alizarin red histology shows bone nodule formation on the scaffolds coated with pPEA + FN + BMP-2 (arrow) after 21 days. Scale bar = 20 µm.

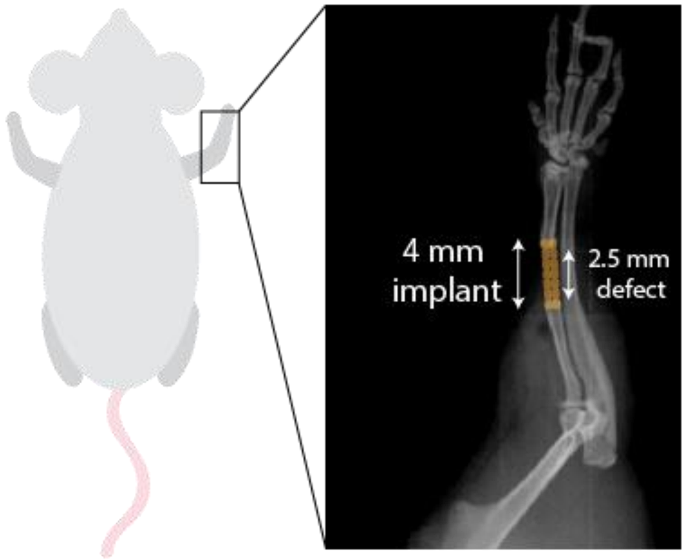

Figure S11. Sketch of the murine radial segmental bone defect. A 4-mm polyimide sleeve was fit between the proximal and distal ends of the 2.5-mm defect. The tube was coated with pPEA, FN, and BMP-2 to enhance osteogenesis.

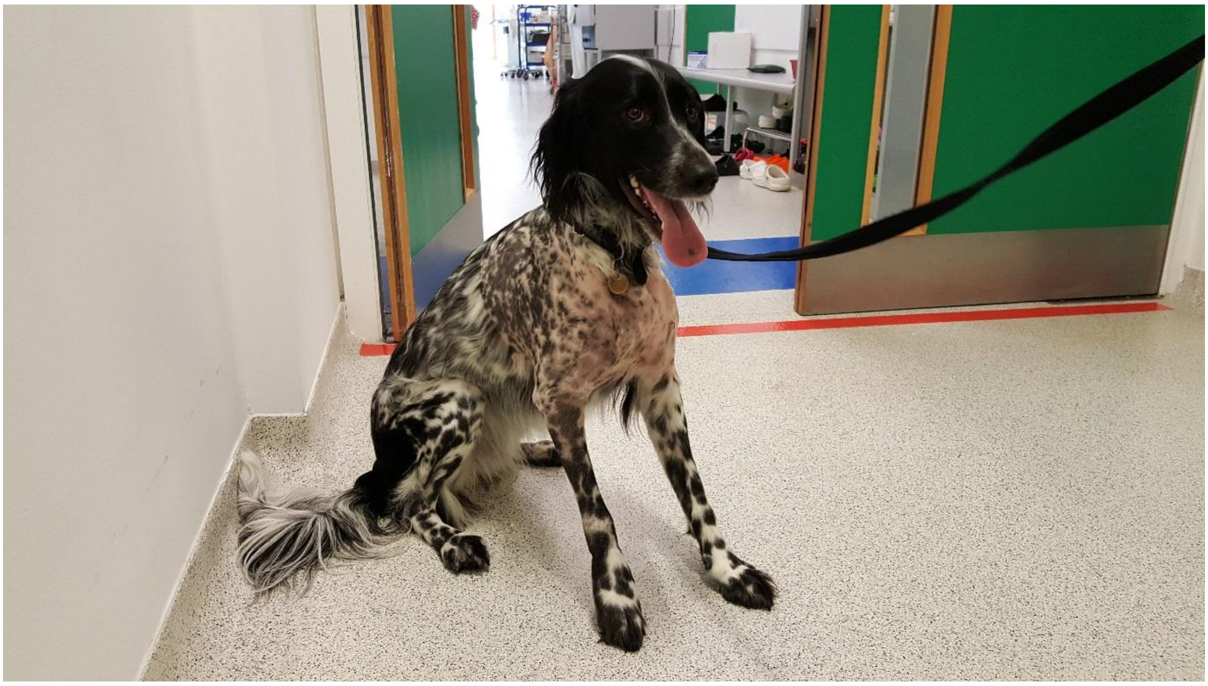

Figure S12. Eva, a two-year-old, neutered, female Münsterländer dog who sustained a fracture of the humerus (right leg) in a road traffic accident. An infected non-union developed and surgery was performed to implant pPEA-chips coated with FN and BMP-2 within the fracture gap. By five months post-operatively, the fracture had healed, the dog had resumed normal exercise, and there was no clinical evidence of a recurring infection.

Video S1. Video of Eva, a two-year-old, neutered, female Münsterländer dog who sustained a fracture of the humerus (right leg) in a road traffic accident. An infected non-union developed and surgery was performed to implant pPEA-chips coated with FN and BMP-2 within the fracture gap: 7 weeks after the surgery (May 2017) and 18 months after the surgery (October 2018).
